# Assessing the Capacity of a Denoising Diffusion Probabilistic Model to Reproduce Spatial Context

**DOI:** 10.1109/TMI.2024.3414931

**Published:** 2024-10-28

**Authors:** Rucha Deshpande, Muzaffer Özbey, Hua Li, Mark A. Anastasio, Frank J. Brooks

**Affiliations:** Department of Biomedical Engineering, Washington University in St. Louis, St. Louis, MO 63130 USA; Department of Electrical and Computer Engineering, University of Illinois Urbana–Champaign, Urbana, IL 61801 USA; Department of Bioengineering, University of Illinois Urbana–Champaign, Urbana, IL 61801 USA, and also with the Department of Radiation Oncology, Washington University School of Medicine, St. Louis, MO 63110 USA; Department of Bioengineering, University of Illinois Urbana–Champaign, Urbana, IL 61801 USA; Center for Label-Free Imaging and Multiscale Biophotonics (CLIMB), University of Illinois Urbana–Champaign, Urbana, IL 61801 USA

**Keywords:** Denoising diffusion probabilistic models, deep generative model evaluation, medical image synthesis, stochastic context models, stochastic object model

## Abstract

Diffusion models have emerged as a popular family of deep generative models (DGMs). In the literature, it has been claimed that one class of diffusion models—denoising diffusion probabilistic models (DDPMs)—demonstrate superior image synthesis performance as compared to generative adversarial networks (GANs). To date, these claims have been evaluated using either ensemble-based methods designed for natural images, or conventional measures of image quality such as structural similarity. However, there remains an important need to understand the extent to which DDPMs can reliably learn medical imaging domain-relevant information, which is referred to as ‘spatial context’ in this work. To address this, a systematic assessment of the ability of DDPMs to learn spatial context relevant to medical imaging applications is reported for the first time. A key aspect of the studies is the use of stochastic context models (SCMs) to produce training data. In this way, the ability of the DDPMs to reliably reproduce spatial context can be quantitatively assessed by use of post-hoc image analyses. Error-rates in DDPM-generated ensembles are reported, and compared to those corresponding to other modern DGMs. The studies reveal new and important insights regarding the capacity of DDPMs to learn spatial context. Notably, the results demonstrate that DDPMs hold significant capacity for generating contextually correct images that are ‘interpolated’ between training samples, which may benefit data-augmentation tasks in ways that GANs cannot.

## I. INTRODUCTION

Deep generative models (DGMs) have shown tremendous potential for advancing medical imaging research [1], [2], [3], [4], [5], [6], [7], [8], [9], [10]. Significant advancements in DGMs have been achieved in the last few years [11], [12], [13], [14], [15]. Recently, a novel paradigm based on diffusion generative modeling [16] has been actively developed and explored in medical image research [3], [4], [5], [17]. Three related formulations appear in the diffusion modeling literature: denoising diffusion probabilistic models (DDPMs) [18], [19], [20], score-based generative models (SGMs) [12], and stochastic differential equations (SDEs) [13]. The first formulation, DDPM [18], [19], [20], is chosen as the focus of this work. DDPMs are designed to estimate the probability density function of the target data distribution by learning to translate an initial noise distribution to the target data distribution over multiple intermediate steps that are modeled as probabilistic transitions. Furthermore, they have been employed for medical imaging applications ranging from medical image synthesis [3], [4], [5], [17], [21] to image reconstruction [14], [22], [23], [24].

It has been claimed that DDPMs demonstrate superior performance in medical image synthesis as compared to generative adversarial networks (GANs) [5], [17], [21], [25], [26]. However, this claim was based on comparisons that primarily relied on ensemble-based evaluation measures employed in natural image feature spaces such as Fréchet Inception Distance (FID) [27], precision and recall [28], or conventional image quality measures such as the structural similarity index measure (SSIM) [29]. The relevance of these evaluation measures to medical image assessment has received limited attention [30], and thus the relevance remains largely unknown. Similarly, other evaluation approaches developed primarily for natural images [31], [32], [33], [34] also may not be sufficient for assessing downstream utility in medical imaging tasks. On the other hand, visual evaluation of medical images [35], [36] is challenged by the need for domain expertise. Thus, although innovations in DGMs have been translated rapidly from computer vision to medical imaging research, the strategies employed to evaluate DGMs are rarely tailored for medical imaging applications [1], [2], [3] and there remains an urgent need for an objective evaluation of diffusion models to determine their applicability for biomedical imaging.

In this work, the suitability of a DDPM for medical imaging applications is tested via its capacity to reproduce prescribed spatial context, represented via certain per-image statistics. Our evaluation approach is similar to some previous works [37], [38]. Here, “context” is defined as domain-relevant information. Context cannot necessarily be learned from just one image, but can be explicitly encoded, or implicitly emergent. Examples are: the prevalence of image classes (i.e., medical conditions) in an ensemble, the size and shape of specific organs, anatomical constraints such as the number of ribs or *relative* size/location of organs, or the organ-specific appearance of image texture. Contextual errors have previously been reported in GAN-generated ensembles [21], [37], [38], [39], but their occurrence in DDPM-generated ensembles has not been studied. Assessing the reproducibility of context [40], [41], [42] provides one way of gaining insights into the performance of DDPM-generated ensembles for medical imaging applications.

Specifically, a DDPM was first tested for its capacity to reproduce *explicit* context via a previously established test bed of stochastic context models (SCMs) [37]. The SCMs represent attributes relevant to medical imaging, in a readily interpretable manner and without anatomical constraints. Next, the DDPM was tested for its capacity to reproduce *implicit* context via an adapted version of a previously published stochastic object model (SOM) that describes anatomical constraints [43]. Per-image, contextual errors in DDPM-generated ensembles were then quantified to provide a measure of the potential suitability of the DDPM to medical imaging tasks involving similar contextual attributes. This evaluation approach can be employed to gain insights into other DGM formulations as well.

## II. Background

### A. Denoising Diffusion Probabilistic Models (DDPM)

In the DDPM framework [20], a small quantity of Gaussian noise is gradually injected into an input image (sampled from a real data distribution) $x_{0}\sim q\left(X_{0}\right)$ over t time steps to eventually obtain the degraded image $x_{t}$. Over a sufficiently large number of time steps T, a sample $x_{T}$ from a Gaussian distribution can be produced. The forward diffusion process is formulated as a Markov chain where the $x_{t}$ and $x_{t-1}$ are related by the transition rule defined as:

(1) $$x_{t}=\sqrt{1-\beta_{t}}x_{t-1}+\sqrt{\beta_{t}}\epsilon ,\;\;\epsilon \sim 𝒩(0,I)$$

(2) $$q\left(x_{t}|x_{t-1}\right)=𝒩\left(x_{t};\sqrt{1-\beta_{t}}x_{t-1},\beta_{t}I\right).$$

Here, 𝒩(0,I) is a Gaussian distribution with zero mean and identity covariance $I,\;\beta_{t}$ is a parameter controlling the addition of noise, and ϵ is additional noise. The reverse diffusion process that maps $x_{T}$ to $x_{0}$ is also formulated as a Markov chain, where each step represents an incremental denoising of the data. The reverse transition probability between $x_{t-1}$ and $x_{t}$ can be represented by a Gaussian distribution for a large T and small $\beta_{t}$:

(3) $$q\left(x_{t-1}|x_{t}\right)\;:=𝒩\left(x_{t-1};\;\mu \left(x_{t},\;t\right),\;\Sigma \left(x_{t},\;t\right)\right).$$

Within each reverse diffusion step, the same neural network with time embedding is employed to approximate the reverse mapping by predicting the mean (μ) and covariance (Σ). The variational lower bound ($L_{vb}$) was employed in the loss function to minimize the negative log-likelihood:

(4) $$L_{vb}=E_{q\left(x_{0:T}\right)}\left[log\frac{q\left(x_{1:T}|x_{0}\right)}{p_{\theta }\left(x_{0:T}\right)}\right]\geq -E_{q\left(x_{0}\right)}\left[\log\;p_{\theta }\left(x_{0}\right)\right],$$

where $p_{\theta }$ is the network parameterization for the approximated reverse diffusion process, θ represents the network parameters, and $E_{q}$ represents expectation over q. The collection of data samples between time steps 0 and T is represented by $x_{0:T}$, while the image samples between time steps 1 and T conditioned on the sample at time step 0 are represented by $x_{1:T}|x_{0}$. The bound can be reformulated as [20]:

(5) $$\begin{array}{l} L_{vb}=log\;p_{\theta }\left(x_{0}|x_{1}\right)\\ \;-\sum_{t=1}^{T}\;KL\left(q\left(x_{t-1}|x_{t},x_{0}\right)\;\|\;p_{\theta }\left(x_{t-1}|x_{t}\right)\right), \end{array}$$

where KL denotes Kullback-Leibler divergence, and $KL\left(q\left(x_{T}|x_{0}\right)\;\|\;p\left(x_{T}\right)\right)$ is omitted as it does not depend on θ. With the reparameterization of the second term in (5), the employed network can predict $\epsilon_{t}$. In Ref. [20], the mean and covariance were learned jointly:

(6) $$\begin{array}{l} KL\left(q\left(x_{t-1}|x_{t},x_{0}\right)∥p_{\theta }\left(x_{t-1}|x_{t}\right)\right)\\ \;=\frac{1}{2{∥\Sigma_{\theta }∥}_{2}^{2}}{∥\mu \left(x_{t},x_{0}\right)-\mu_{\theta }\left(x_{t},t\right)∥}^{2}\\ \;=\frac{{\left(1\;-\;\alpha_{t}\right)}^{2}}{2\alpha_{t}\left(1\;-\;{\overset{‾}{\alpha }}_{t}\right){∥\Sigma_{\theta }∥}_{2}^{2}}{∥\epsilon_{t}-\epsilon_{\theta }\left(x_{t},t\right)∥}^{2}. \end{array}$$

### B. Description of the Stochastic Context Models (SCMs)

The following subsections provide brief descriptions of the three SCMs employed in this work. Each SCM encodes a different set of contextual constraints (see Table I), and is either single-, or multi-class. Further details can be found in a previous work [37], and the data are publicly available [44]. A realization from any SCM is a 256 × 256, grayscale image. The single-class ensemble contained 131072 images, and the multi-class ensembles contained 65536 images per class.

#### 1) Alphabet SCM (A-SCM):

The A-SCM represents prescribed contextual constraints of prevalence and relative positions of features. This SCM is analogous to anatomical scenarios that exhibit constraints in per-image feature prevalence and relative positions of features. A realization of the A-SCM corresponds to a regular grid of 32 × 32-pixel tiles t; each tile represents a letter in the alphabet A={H,K,L,V,W,X,Y,Z}. The per-image frequency of occurrence of all letters was prescribed such that each realization I consisted of the exact set of letters: B={24×H,2×K,16×L,1×V,1×W,8×X,8×Y,4×Z}. That is, a realization can be written as:

(7) $$I=\{t_{r,c}\;:\underset{r,c}{⋃}\;f\left(t_{r,c}\right)=B\},$$

where f(t) represents a template-matching operation that maps an input tile to a letter, and r, c respectively indicate the row and column indices within the grid. The placement of specific letters in a realization I was not constrained, thus creating diverse realizations. However, contextual rules of per-image prevalence of letters as well as of letter-pairs were always upheld in each realization. The following rules of conditional letter-pair prevalence were prescribed:

(8) $$\begin{array}{l} p\left(f\left(t_{r,c+1}\right)\right.\left.=Y|f\left(t_{r,c}\right)=X\right)=1,\\ \;p\left(f\left(t_{r,c}\right)\right.\left.=Z|f\left(t_{r+1,c}\right)\in \{V,W,K\}\right)=1. \end{array}$$

Each realization included four ordered letter pairs: X-Y (horizontal), Z-K, Z-V, and Z-W (vertical). The respective per-image prevalences of these letter-pairs were 8, 2, 1, and 1. Note that the letters in these pairs always occurred only as a part of the prescribed letter-pair. Post-hoc classification of letters in DGM-generated ensemble was performed via template matching.

#### 2) Voronoi SCM (V-SCM):

The four-class V-SCM represents joint contextual constraints in prevalence, intensity and texture. This SCM is logically analogous to microscopy images that contain distinct cells differing in their intensity, texture, morphology, and prevalence. Specifically, each realization I can be represented as a set V, which consists of Voronoi regions $v_{i}$ and edges $e_{i}$. Here, i={1,2,...,c}, where cϵ{16,32,48,64} denotes the class of an image as well as the number of Voronoi regions in an image. The placement of region centers within I was spatially random, introducing positional variance. By setting edges e to an intensity level of 0, the recoverability of Voronoi regions was enhanced. All pixels in a region $v_{i}$ were allocated a constant grayscale intensity g chosen from a set of 128 predefined values between 1 and 254. Furthermore, the grayscale intensity of a region was perfectly rank-correlated with its area: $\rho \left(area\left(v_{i}\right),g\right)=1$, where ρ is the Spearman rank-order correlation coefficient. Post-hoc processing of generated realizations to extract individual Voronoi regions involved edge detection via skeletonization and Sauvola thresholding [45]. The error in region detection was not large enough to affect the inferences in our experiments.

#### 3) Flag SCM (F-SCM):

The eight-class F-SCM represents joint contextual constraints in prevalence, intensity, texture, and absolute position; this SCM is logically analogous to the joint constraints specific to organs. Each image I, in class c, was divided into a 16 × 16 grid of tiles, where each tile represented either the foreground $\left(f_{k}\right)$ or the background $\left(b_{k}\right)$, and k denoted the position of the tile within the grid. Thus, each image, irrespective of class, consisted of exactly the same number of foreground and background tiles $I_{c}=\left\{80\times f_{k},176\times b_{k}\right\}$— this removed the zero-order variance in the number of foreground pixels across classes. A realization *I* was modeled as:

(9) $$I_{c}=\sum_{k}\left(a_{kc}f_{k}+\left(1-a_{kc}\right)b_{k}\right),$$

where $𝒜\in {\left\{0,\;1\right\}}^{K\times C}$ is a binary matrix signifying background (0) or foreground (1) for all K tile indices in C classes. As a result, the image class is one of eight different foreground arrangements, and A denotes the pre-defined, class-specific foreground patterns. Intensity distributions for $f_{k}$ and $b_{k}$ were prescribed as:

(10) $$\begin{array}{l} f_{k}\sim 152X+96,\;\text{where}\;X\sim Beta(\alpha =4,\beta =2),\\ b_{k}\sim 192X+8,\;\text{where}\;X\sim Beta\left(\alpha =2,\beta =4\right). \end{array}$$

The respective variates were placed randomly in the foreground $\left(f_{k}\right)$ and background $\left(b_{k}\right)$. The presence of multiple constraints not only enabled the testing of the reproducibility of joint constraints at once, but also enabled the testing of preferential learning of any of these constraints. Additionally, a set of 24 tile-location indices were forbidden as foreground in any class, this enabled investigation of the nature of interpolation between classes. Comparison of tilewise intensity means to determine foreground followed by mean absolute error computation against the expected foreground pattern yielded class identity for the DGM-generated ensemble.

### C. Description of the Adapted VICTRE Stochastic Object Model (VT-SOM)

The anatomically realistic stochastic model: VT-SOM, is an adapted version [46] of a four-class SOM [43] that describes the anatomy of the human female breast. A brief description of the adaptation procedure follows. First, 8000 realizations of 3D voxelized maps of human numerical breast phantoms were generated and all tissues were allocated linear attenuation co-efficients corresponding to x-rays at energy 30 keV. Fifteen coronal slices were extracted from approximately the central third of each phantom volume and the resulting images were then downsampled to 512 × 512 while retaining the ligament skeleton. Finally, the tissue types were allocated values in the 8-bit grayscale range largely consistent with their linear attenuation co-efficients. The VT-SOM comprised four breast types – fatty, scattered, heterogeneous, and dense – based on the Breast Imaging Reporting and Data System (BI-RADS) [47] classification of breast density. The dataset was then cleaned to eliminate slices with incomplete breast regions and the resulting ensemble of 108,000 slices from the VT-SOM was employed to train DGMs. The VT-SOM has a class prevalence (as determined by the relative proportion of fatty to glandular tissue) in the ratio 1:4:4:1 for the fatty, scattered, heterogeneous, and dense breast types. This ratio is based on the approximate population prevalence [47].

## III. Methods

### A. Network Trainings

Four of the most widely used DGMs were employed in this work. These included two popular diffusion models: Denoising Diffusion Probabilistic Model (DDPM) [20] and MedFusion [21] a kind of a latent diffusion model (LDM), and two popular GAN architectures: StyleGAN2 (SG2) [15] and StyleGAN-XL (SG-XL) [48]. StyleGAN2 was chosen as a representative GAN model because (i) it is a modern GAN that does not incorporate modifications aimed at improving performance on the ImageNet dataset, and (ii) it is currently a popular backbone for GAN-based methods due its stability and generalizability to datasets that are not rotationally or translationally invariant. Diffusion generative models and generative adversarial networks (GANs), both, are known to yield high quality image as compared to other DGM families, such as variational autoencoders (VAEs) and normalizing flows [49]. Hence, the latter two DGM families are excluded in this work while GANs are included to provide a high bar for comparison to diffusion models. Two of the four DGMs: DDPM and SG2, representing the two chosen DGM families, were trained on all datasets described in Sec. II; the other two DGMs: SG-XL and LDM, served as additional baselines for the V-SCM and VT-SOM datasets. Note that different DGMs have different recommended training strategies and optimal training parameters. Because the recommended default hyperparameters have been optimized for visual quality and Frechet Inception Distance (FID) scores, the defaults were used for all trainings unless specified otherwise. For a fair comparison, each DGM was trained with a fixed compute budget for a certain image size. This budget was 900 GPU-hours on a Nvidia GTX TitanX GPU for all SCMs (image size 256 × 256), and 900 GPU-hours on a Nvidia Quadro RTX 8000 for the VT-SOM (image size 512 × 512).

All DDPM models were trained with the recommended architectural improvements [20]. Furthermore, two variants of DDPM: class-conditioned DDPM, and foundational DDPM were also trained/fine-tuned to assess if these variations improved the performance of DDPM. The class-conditioned DDPM was trained on the two multi-class SCMs: V-SCM and F-SCM. Foundational DDPM was fine-tuned for V-SCM, and the more complex VT-SOM dataset. In case of the VT-SOM, the foundational DDPM available for image size 512 × 512 was modified to bypass class conditioning so that this model could be employed for unconditional image generation. All other training parameters were retained as default for both, class-conditioned and foundational DDPMs. The second diffusion model, LDM, consisted of training a VAE on the original images, followed by training a DDPM on the latent representation of the original images encoded by the trained VAE. The default dimensions of the latent representation were retained for VT-SOM (8 × 128 × 128), and lowered to (3 × 64 × 64) for V-SCM, which consisted of simpler and smaller images as compared to VT-SOM. For the first GAN: SG2, config-e was employed for all SCMs, and configf was employed for VT-SOM with a lower than default latent space size of 256 to improve training stability. SG-XL models were trained progressively as recommended, from a resolution of 32 × 32 to the image size, with an upscaling factor of 2. The total compute budget over all resolutions was fixed; the DGM trained for 5 million images at the last two resolutions, and 1 million images at each of the lower resolutions based on the recommended settings as well as our empirical findings. In all cases, the last model was chosen except in case of SG2 trained on the VT-SOM, where the model with the least FID was chosen for further analysis.

A four-class classifier with a VGG-16 backbone [50] was also trained on a distinct ensemble obtained from the VT-SOM to predict the classes in the training dataset. The training and validation datasets consisted of 5000, and 1500 images per class, respectively. The classifier was trained for 400 epochs and the model corresponding to the least validation loss was chosen for inference. The per-class mis-classification rates were observed to be 0%, 0.07%, 0.37%, 0.87% in a test set of 3000 realizations for each of the four classes.

### B. Methods: Evaluation Framework

Evaluations for studies involving the SCMs were performed after appropriate post-hoc analysis of the DGM-generated images, as described in a previous work [37]. For the VT-SOM study, first, individual tissues were segmented via global thresholding based on the prescribed tissue-specific intensity distributions. Next, the following feature types were extracted from all training and generated images: skeleton statistics [51], morphological features [52], texture features [53], and the ratio of fatty tissue to glandular tissue (F/G ratio) [47]. Principal component analysis was performed on (i) each of these feature families separately (e.g., only skeleton statistics, only morphological features) and (ii) all features together, and the top 10 principal components were retained. The cosine similarity was computed for 10,000, 10-dimensional, randomly selected training-training pairs; this produces an estimated distribution of the similarity among realizations. A second distribution was computed for the same number of randomly selected training-generated pairs. The differences between the two distributions was summarized via the Kolmogorov-Smirnov (KS) test statistic [54] in each case: (i) individual feature families, and (ii) for all features. For class-wise analysis, class coverage and density [55] were computed in the top two principal component space, while class prevalence was obtained by employing the classifier described in the previous subsection.

## IV. Results

It is noted that the performance of DGMs may vary with the choice of training hyperparameters, or even random initialization. The performance reported in this work is only representative of typically trained models and may not indicate the best performance possible for any DGM; identifying the “best possible” instance of a DGM is a massive computational undertaking, and a fundamentally different problem than the goal of this work.

### A. Results From the A-SCM

**Fig. 3.**
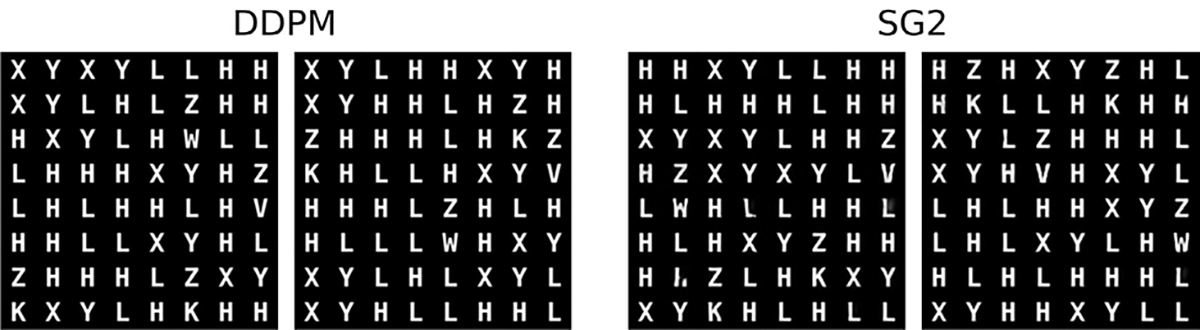
Visually high quality generated samples from the DDPM (left) and SG2 (right).

Sample realizations from all DGMs trained on A-SCM are shown in Fig. 3; high visual quality was observed for instances from all DGMs. The FID-10k values were: 0.1 (DDPM), and 6.7 (SG2).

Only realizations within which all letters were visually recognizable were included for further analysis. Recognition was automated via a pattern match filter [37]; the rejection threshold was set to correspond with unambiguous, visually sharp letters as observed in generated images. All DDPM realizations exhibited only recognizable letters, but only 59% of the SG2 realizations were completely recognizable. Ten-thousand, well-formed realizations from each model were selected randomly for further analysis. Single letter prevalence was assessed via a chi-squared goodness-of-fit test with the critical value set to 95%. About 99% of all DGMs realizations were acceptable, however, only 0.08% SG2 realizations exhibited perfect letter prevalence; this is in stark contrast to the 98% DDPM realizations exhibiting perfect prevalence.

**Fig. 4.**
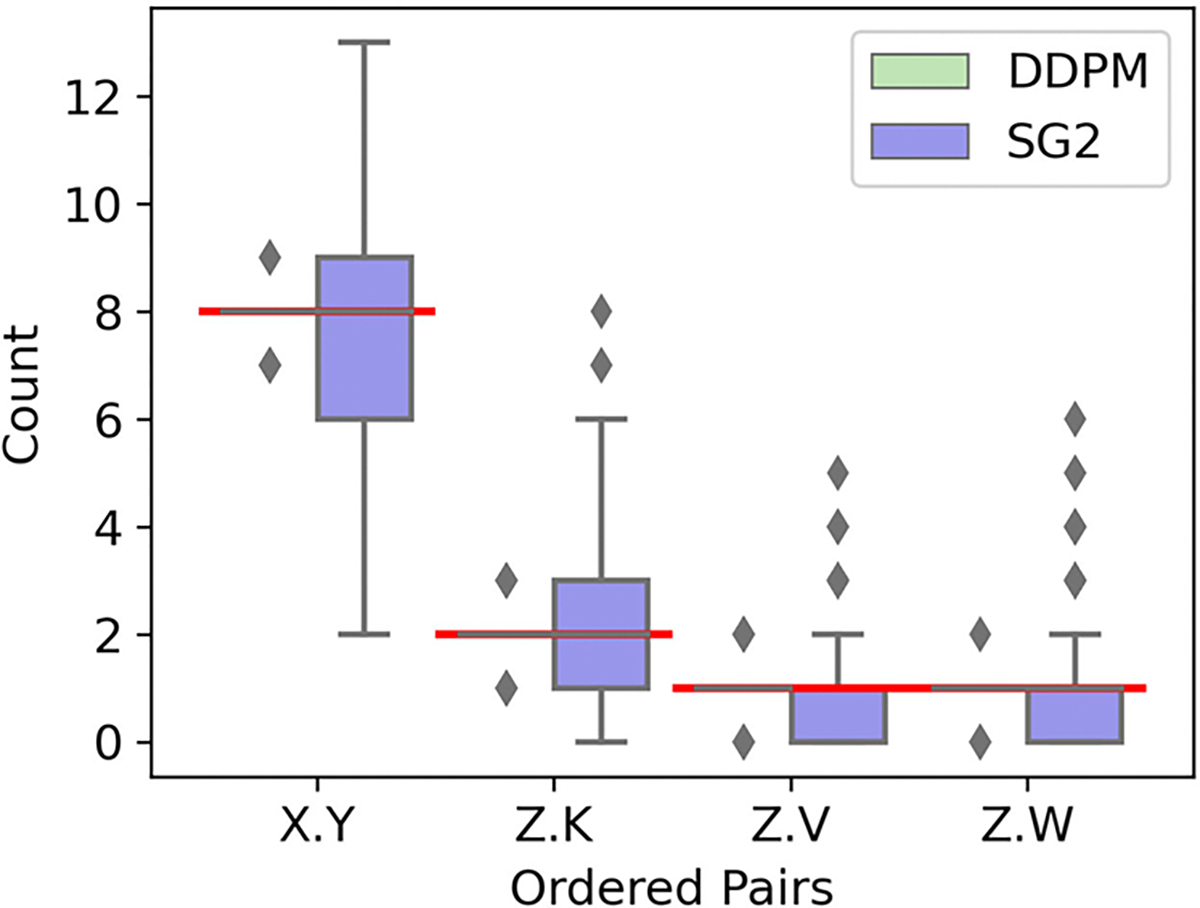
Results from A-SCM. Not only does DDPM clearly outperform SG2, but also achieves near perfect per-image prevalence of the letter-pairs representing contextual constraints. Correct prevalence is marked in red. Note that the DDPM quartiles perfectly overlap with the correct prevalence (in red), and hence, are not visible.

**Fig. 5.**
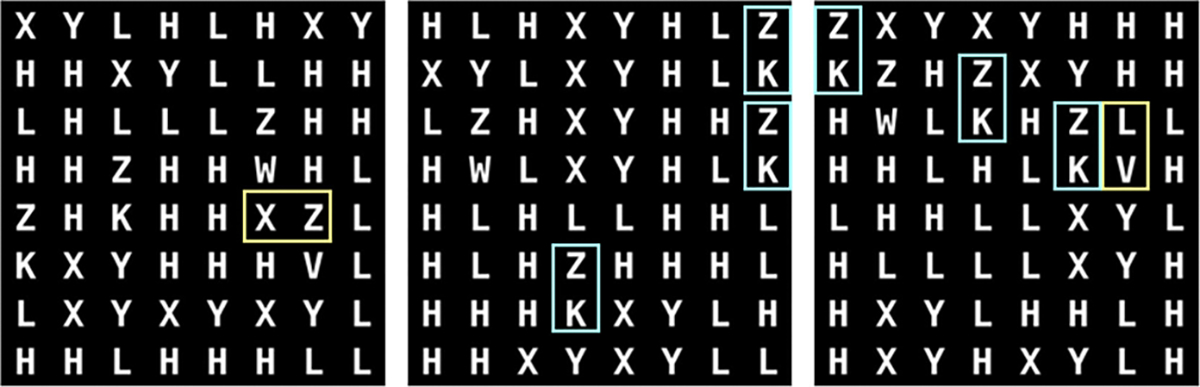
Contextual errors were observed in some DDPM-generated realizations from A-SCM. These manifested as incorrect pairings of letters (yellow) or incorrect per-image prevalence of letter-pairs (blue). In the training data, the letter-pairs X-Y, and Z-V were always in order, and the letter-pair Z-K occurred exactly twice in each image.

Results from the reproducibility of feature-pair prevalences (see Fig. 4) strengthen this finding. All four letter-pairs prescribed in the training dataset were almost perfectly replicated throughout the DDPM-generated ensemble, but most SG2-realizations exhibited substantially incorrect letter-pair prevalences. Some errors in the DDPM realizations are shown in Fig. 5. These examples demonstrate that DDPM occasionally creates new pairings, or displays pairings too frequently, even when realizations are otherwise excellent. Thus, by analogy, an ensemble of biomedical images could appear perfect via spot-checks, and pass traditional tests of distribution similarity, but still include images that are anatomically nonsensical.

### B. Results From the V-SCM

**Fig. 6.**
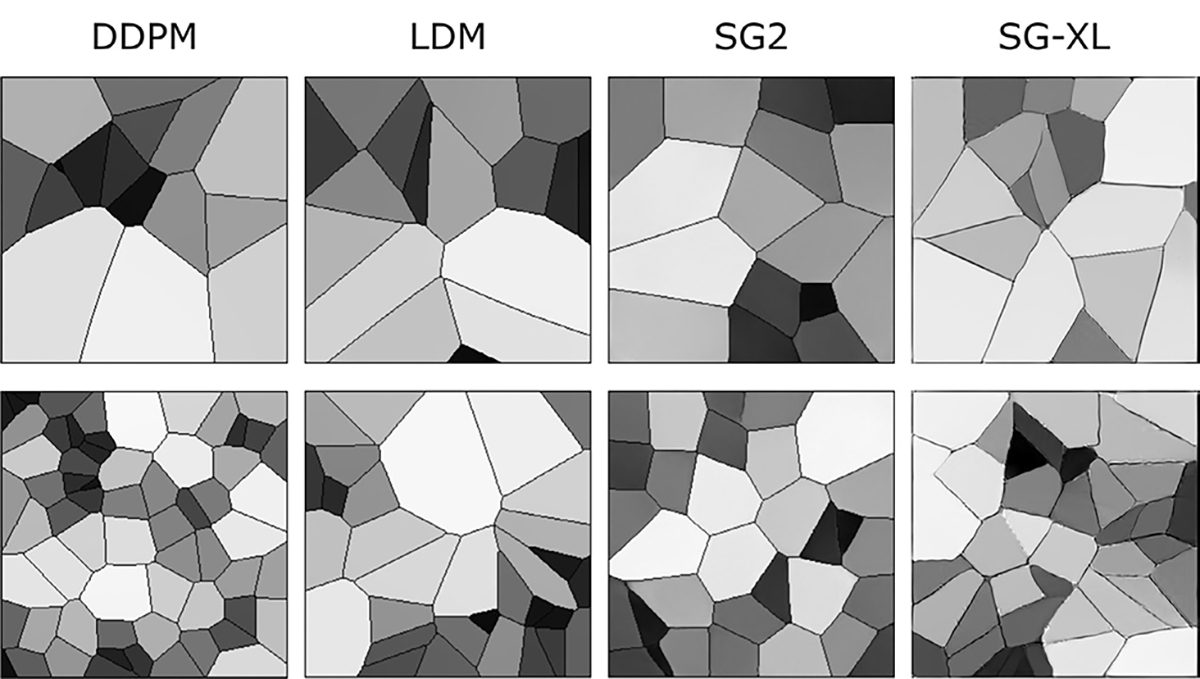
Sample DGM-generated images from the V-SCM. Except for SG-XL, all other DGMs demonstrate visually high quality images.

Samples from DGM-generated ensembles are shown in Fig. 6. High visual similarity was observed for samples from DDPM, LDM, and SG2, whereas SG-XL-generated images demonstrated obvious errors in edge formation. The corresponding FID-10k values were: 1.5 (DDPM), 14.1 (LDM), 25.1 (SG2), 50.5 (SG-XL). Potential reasons for low image quality of SG-XL images generated from V-SCM (and VT-SOM) are discussed in the Discussion section.

DGM-generated images corresponding to the V-SCM were tested for (i) *explicit* contextual rules of shading and prevalence prescribed in the training ensemble, as well as (ii) certain *implicit* contextual features that emerge as a result of the stochastic processes defined in the SCM.

**Fig. 7.**
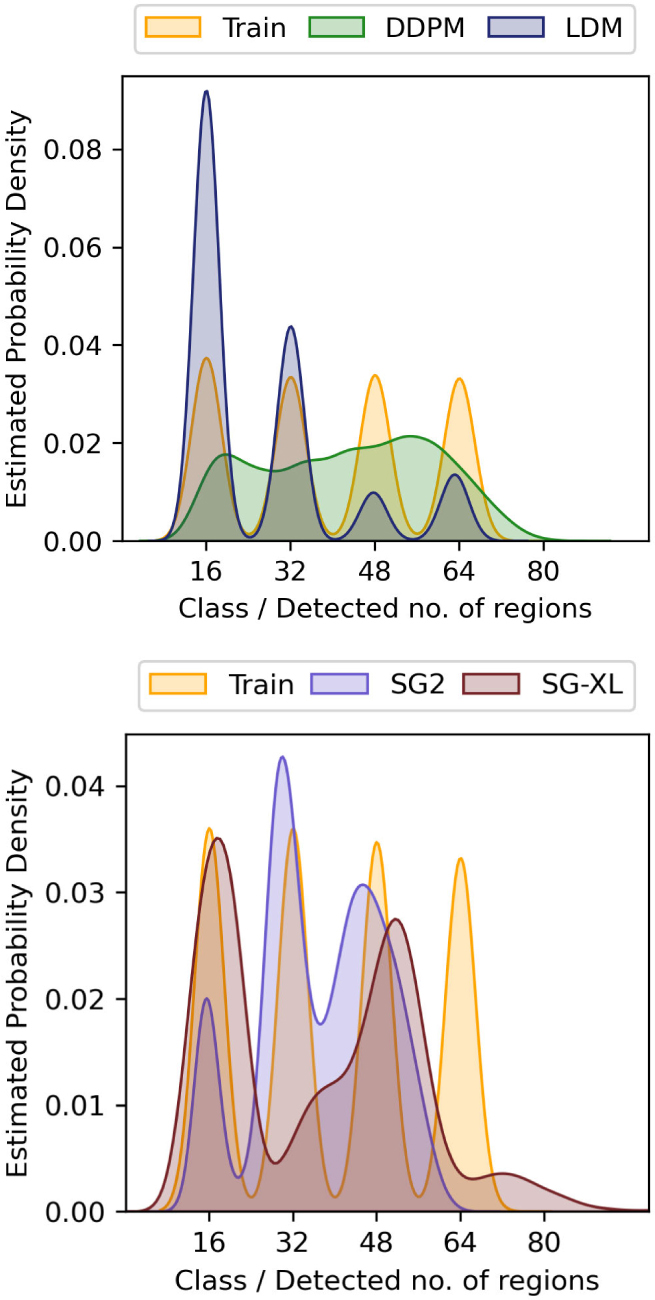
Class-prevalence results from the V-SCM demonstrated via kernel density estimates (KDE) of the data. All DGMs fail to replicate the prescribed uniform class prevalence. The DDPM and SG2 demonstrate interpolation between the four distinct classes in the training dataset. In addition, DDPM also extrapolates beyond the extreme class (class 64) generating realizations corresponding to class 80, which was absent in the training dataset. Although LDM retains the distinct modes in the data, the uniform class prevalence is not respected.

The prescribed perfect correlation between grayscale intensity and area, within each image was observed to be lower in all DGM-generated ensembles. Approximately 4%, 0.1%, 13.7%, and 98% of the DDPM, LDM, SG2 and SG-XL ensembles respectively demonstrated a Spearman rank-correlation *ρ* < 0.9, indicating that the quantitative value of these realizations is partially lost. Next, per-image feature prevalence encoded as the number of Voronoi regions in an image was tested. Recall that, here, the number of regions defines class (see Sec. II–B.2 for class prediction on DGM-generated images). It was observed that no DGM reproduced the prescribed uniform class prevalence exactly (see Fig. 7), although both diffusion models demonstrated good mode coverage, and LDM retained the distinct modes in the training data.

DDPM and SG2 were observed to interpolate between modes such that a substantial number of generated realizations are not any of the classes seen in the training data (see Fig. 7). The extent of interpolation was *less* for SG2, but still non-negligible. The SG2 model did not extrapolate beyond the extreme classes, but the DDPM unequivocally did (see Fig. 9 bottom row). These observations could imply that interpolation and extrapolation are functionally equivalent within the two DGMs. In case of SG-XL, the apparent class extrapolation was a result of badly formed skeletons, as observed in Fig. 6, unlike DDPM where class extrapolation was demonstrated via visually high quality images.

**Fig. 8.**
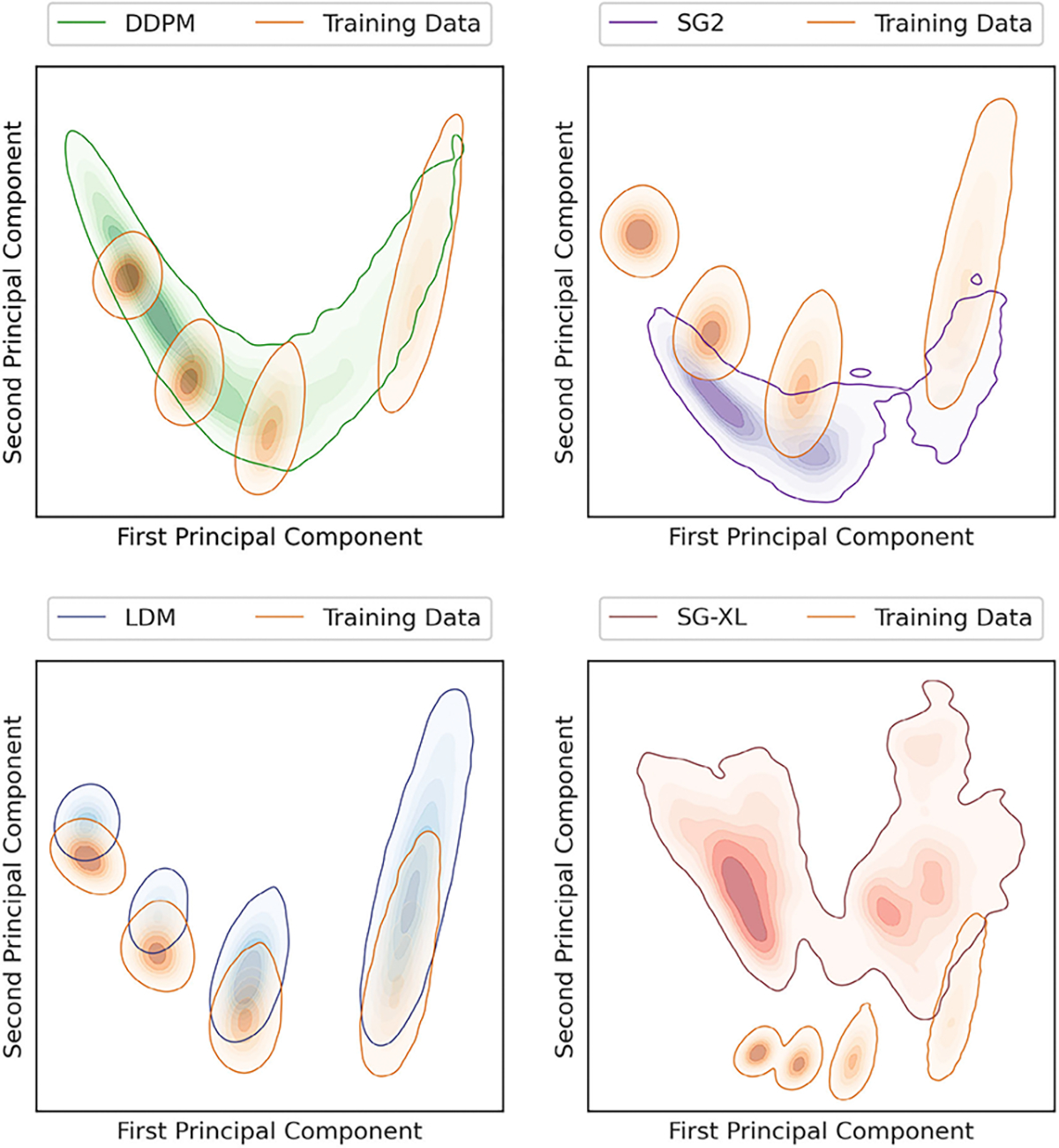
Results from the V-SCM. Principal component analysis (PCA) of the statistics representing implicit context demonstrates that interpolation between classes also resulted in an interpolation of the emergent implicit context in case of the DDPM (top left), but not other DGMs. LDM (bottom left) generally respected the distinct classes and their respective implicit context. The emergent implicit context was not exactly replicated by SG2 and SG-XL (right) as seen in the partial overlap between training and generated data. Note that the PCA plots are represented via kernel density estimation for display.

Interpolation effects were further explored by assessing the implicit context typically arising in Voronoi diagrams [56]. Given that Voronoi diagrams represent a unique solution to the space partitioning problem, variation in one statistic (e.g., number of regions) should affect all correlated statistics if the implicit context specific to Voronoi diagrams is exactly reproduced [56]. The following per-image statistics (computed via a Python package [51]) were chosen to represent implicit context: number of Voronoi regions, number of junctions, junction density, mean and standard deviation of Voronoi edge lengths, mean and standard deviation of the area of a Voronoi region. Results from principal component analysis performed on these statistics are shown in Fig. 8.

**Fig. 9.**
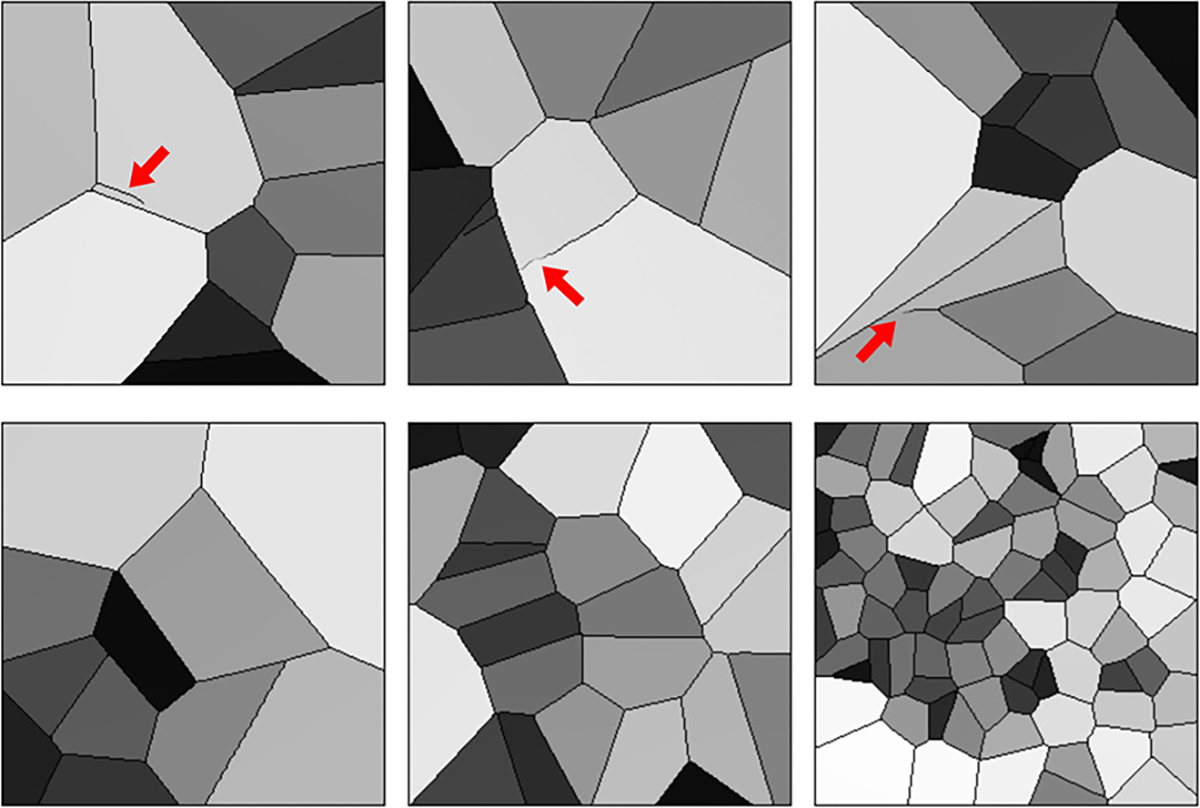
DDPM-generated samples from V-SCM exhibit implicit contextual errors like disjoint Voronoi edges (top row) and explicit errors like incorrect region count (bottom row). Although realizations in the bottom row are visually acceptable, the number of regions per-image indicating class is lower than (left), interpolated between (center), or extrapolated beyond, the classes in the training data (right).

It was observed that although both diffusion models respected class-specific implicit context, DDPM-generated realizations interpolated between classes following the trend in implicit context defined by the training data. This was confirmed by visual spot checks of sample realizations from the new classes and their placement in the PCA plots. This result suggests that the DDPM generated a substantial number of realizations from new classes (via interpolation), but, perhaps more importantly, that those realizations may be genuine Voronoi diagrams. In Sec. V, this result is discussed further. On the other hand, this was not the case for SG2, which may be more prone to errors in implicit context for a similar interpolation between classes, suggesting that at least a fraction of the interpolated SG2 images may not be considered Voronoi diagrams. LDM demonstrated negligible interpolation between classes, and slight extrapolation of each class, suggesting that class identity was strongly captured in the latent representation of the training dataset. Finally, images from SG-XL rarely demonstrated the implicit context expected in Voronoi diagrams. Out of all DGMs employed in this study, only DDPM demonstrated contextually correct class interpolation. However, occasional errors in statistics representing implicit context were visually observed in DDPM-generated images (see Fig. 9), indicating that all implicit contextual features were not always perfectly reproduced. Thus, results from the V-SCM indicate that a large fraction of DDPM-generated images, but not all images, may be contextually correct in terms of quantitative meaning and class identity.

### C. Results From the F-SCM

**Fig. 10.**
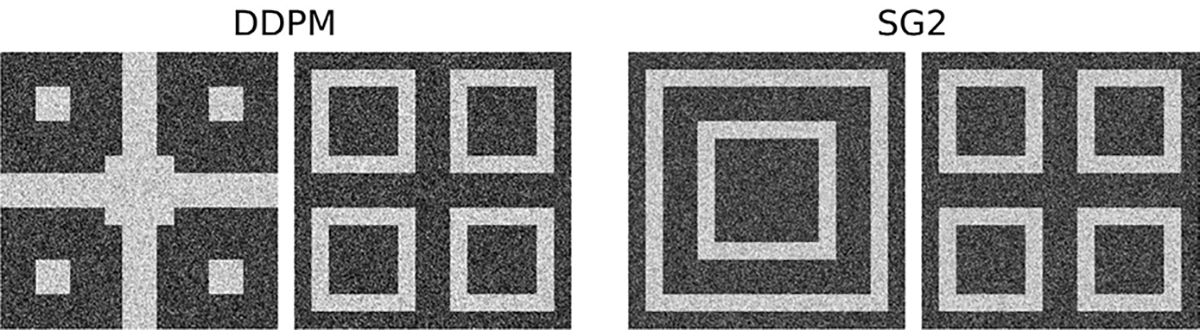
Visually high quality generated samples from the DDPM (left) and SG2 (right) trained on the F-SCM.

Sample realizations with high visual quality from all DGMs trained on the F-SCM are shown in Fig. 10. The FID-10k values were: 5.7 (DDPM), and 25.7 (SG2).

Quantitative results from the F-SCM that encodes joint contextual constraints in per-image feature prevalence, position, grayscale intensity, and texture are given in Table II. The class-specific foreground patterns representing joint constraints in position and prevalence were correctly reproduced by all DGMs for over 98% of the ensemble when tested via foreground template matching and relative mean absolute errors (RMAE). This is also visually evident in the DGM-generated images (see Fig. 10).

**Fig. 11.**
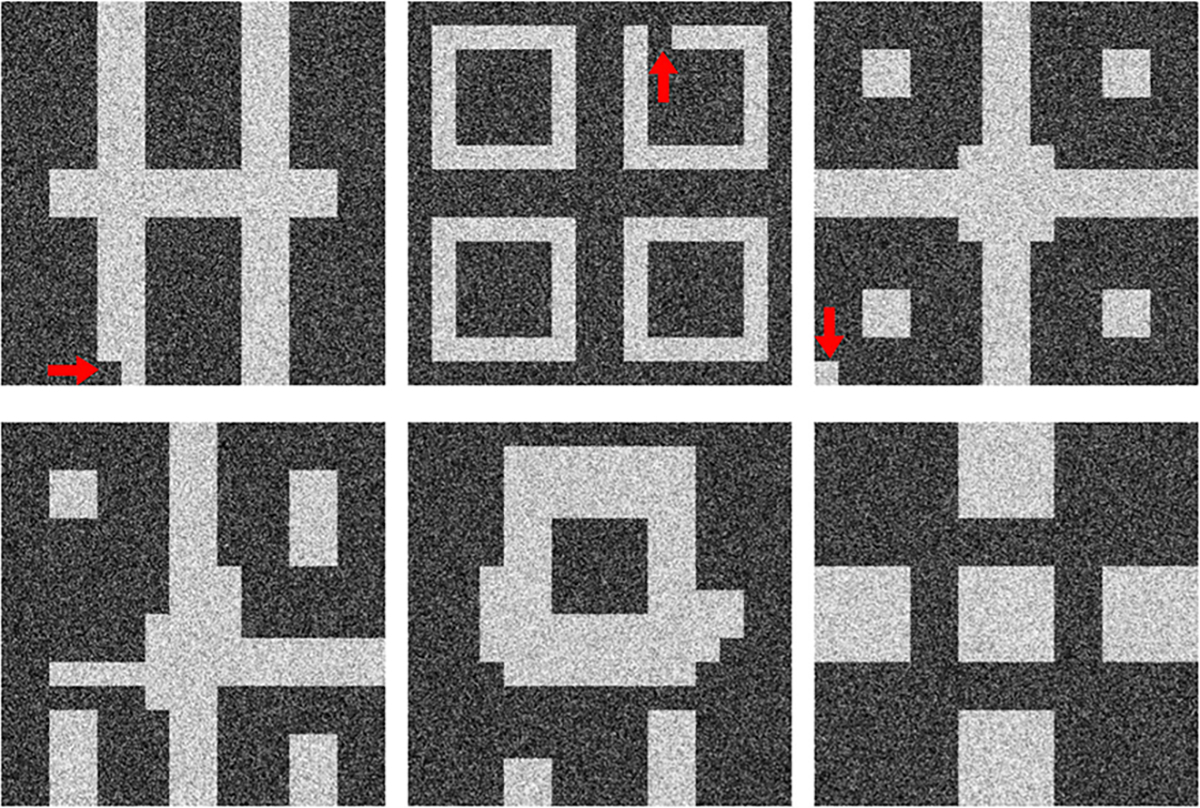
Contextually incorrect DDPM-generated samples from the F-SCM are shown. Top row: Minor errors in the foreground patterns due to a single misplaced tile were observed. Bottom row: Major errors in the class-specific foreground patterns were also observed. None of the foreground patterns in this row were present in the training data.

However, errors in foreground patterns such as those shown in Fig. 11 were observed in about 1% of the DDPM-generated ensemble. This is an important observation and the learning behavior of the DDPM is discussed in detail in Section V. Note that the errors always occurred as misplaced or absent foreground tiles. Additionally, tiles which are never foreground in any class appeared as foreground in 0.1% of the DDPM-generated ensemble, but never in the SG2-generated ensemble. This suggests that the DDPM learned individual motifs that create foreground patterns instead of entire image-level patterns. Texture arising from the randomness in pixel placement was correctly reproduced in over 95% of SG2-generated ensembles and over 99% of DDPM-generated ensembles as measured per tile via Moran’s I [57]. Last, the prescribed per-image intensity distributions as measured via the *χ*^2^ goodness-of-fit test (at 95% critical value) over each image were assessed. Except for a small fraction of SG-2 generated images, virtually all images from all DGMs were beyond the 99.5th percentile of the value of the *χ*^2^ statistic computed on the training data separately for the foreground and background intensity distributions. These results might indicate some difficulty in learning multiple joint contextual constraints at once. Furthermore, the results also highlight the potential of a SCM-based evaluation approach, wherein contextual constraints are progressively added for the assessment of DGMs.

### D. Results From the VT-SOM

**Fig. 12.**
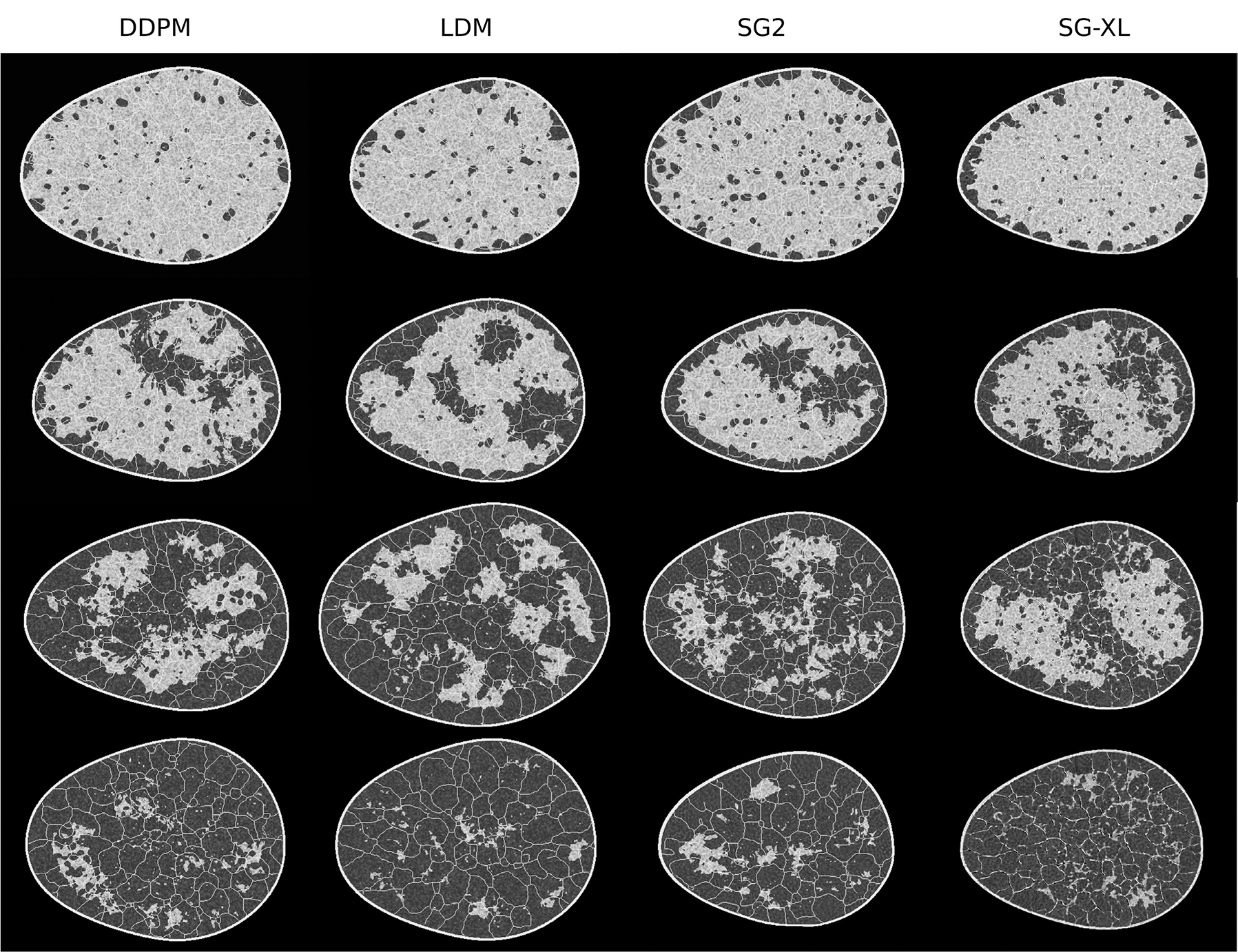
DGM-generated samples with high visual quality, corresponding to all four classes in the VT-SOM are shown. Recall that, here, the fat-to-glandular (F/G) ratio defines class.

Images generated from all DGMs trained on the VT-SOM are shown in Fig. 12. Images from the DDPM, LDM, and SG2 demonstrated high visual similarity with the training data (see Fig. 12) as well as low (<10) FID-10k scores; DDPM-generated images in particular, had distinctly superior visual image quality. Images from SG-XL demonstrated visually obvious artifacts in ligament formation, similar to the artifacts observed in edge formation in V-SCM (Fig. 6). The corresponding FID-10k scores for all DGMs were: 1.3 (DDPM), 14.3 (LDM), 9.8 (SG2), and 30.3 (SG-XL).

Results from the VT-SOM demonstrate that the DDPM clearly outperforms all DGMs on most feature sets (see Table III) included in the study, namely, texture features, morphology features, skeleton statistics, and the ratio of fatty to glandular tissue. (See Sec.III–B for a description of the evaluation framework.) In general, both diffusion models performed better or at least comparable to GANs for most feature sets. Similar trends were observed for all feature sets, with DDPM performing better than LDM, which generally performed better than both GANs. This effect was particularly strong for morphology features (KS statistic values for DDPM, LDM: 0.049, 0.160, and SG2, SG-XL: 0.278, 0.255) and skeleton statistics (KS statistic values for DDPM, LDM: 0.006, 0.109, and SG2: 0.195). Skeleton statistics were not computable for SG-XL images because of major errors in formation that impacted detectability. However, in the reproducibility of F/G ratio (also representative of class), both GANs outperformed both diffusion models, with SG2 demonstrating the best performance. While SG2 demonstrated mode collapse in the F/G ratio and some interpolation between modes, DDPM demonstrated interpolation as well as substantial extrapolation, similar to results from the V-SCM. This result is also reflected in the high F/G fidelity for SG2 but not DDPM, as expected. Some DDPM-samples with extreme F/G ratio, not seen in the training, were also observed; the breast region in these samples seemed to be formed almost entirely of glandular tissue.

**Fig. 13.**
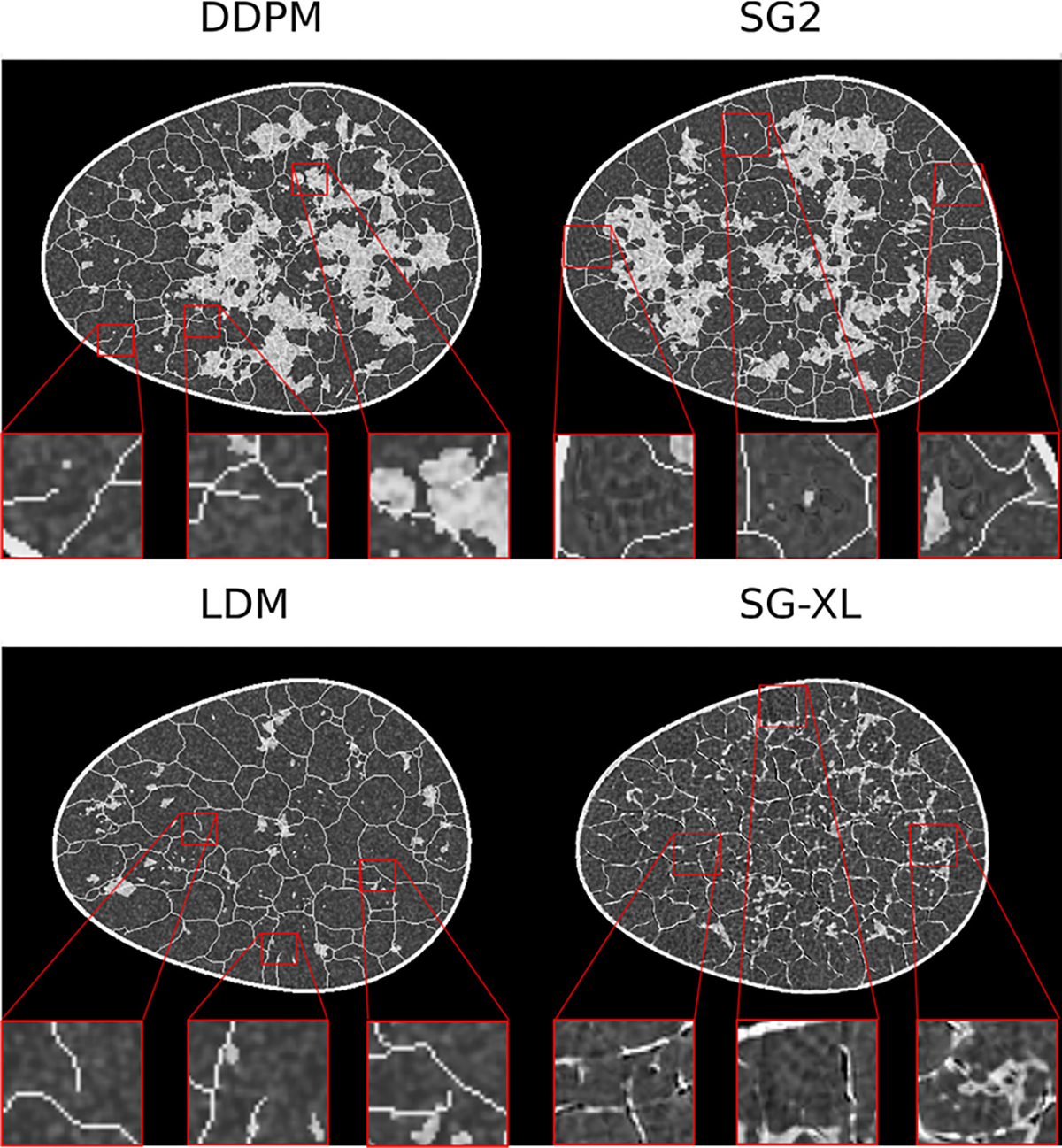
Samples from DGMs trained on VT-SOM show varied artifacts. The DDPM and LDM images (left) exhibit strong visual quality but reveal structural errors like broken ligaments (inset). SG2 and SG-XL images (right) display artifacts in ligament structure, tissue texture, and shading (inset), not present in the training data.

Random samples of 200 images each from the training and DDPM-generated ensembles were visually inspected by non-domain-experts for any immediately obvious errors. Occasional artifacts in ligament structures were visually noticeable in the DDPM-generated images (see Fig. 13). While major breaks in ligaments were observed in 1 in 6 images in the training ensemble, this rate doubled to 1 in 3 images in the DDPM-generated ensemble. These results indicate that even though DDPM outperformed all DGMs, it routinely synthesizes images with anatomical artifacts that can be spotted in the ligament structures even by a non-domain-expert upon casual inspection. This should be taken into account before using the realizations for decision support.

Next, class-wise analysis was performed after predicting class labels on generated ensembles by employing a classifier with a VGG-16 [50] backbone (see Sec. III–A). All four classes from the training ensemble were well represented in the DDPM-generated ensemble, but the other three DGMs demonstrated different kinds of errors (see Table IV). Class coverage and density [55] results demonstrate that the LDM-generated images had moderate to high class density (indicative of class fidelity) and moderate coverage (intra-class diversity) as compared to the training data. SG2 demonstrated superior coverage or density as compared to LDM for some classes, but still ranked lower than DDPM for both these attributes. Possibly the worst performance was demonstrated by SG-XL, with very low class density as well as coverage. Performance of SG-XL is further discussed in the Discussions section.

### E. Results From Variations of the DDPM

#### 1) Class-Conditioned DDPM:

**Fig. 14.**
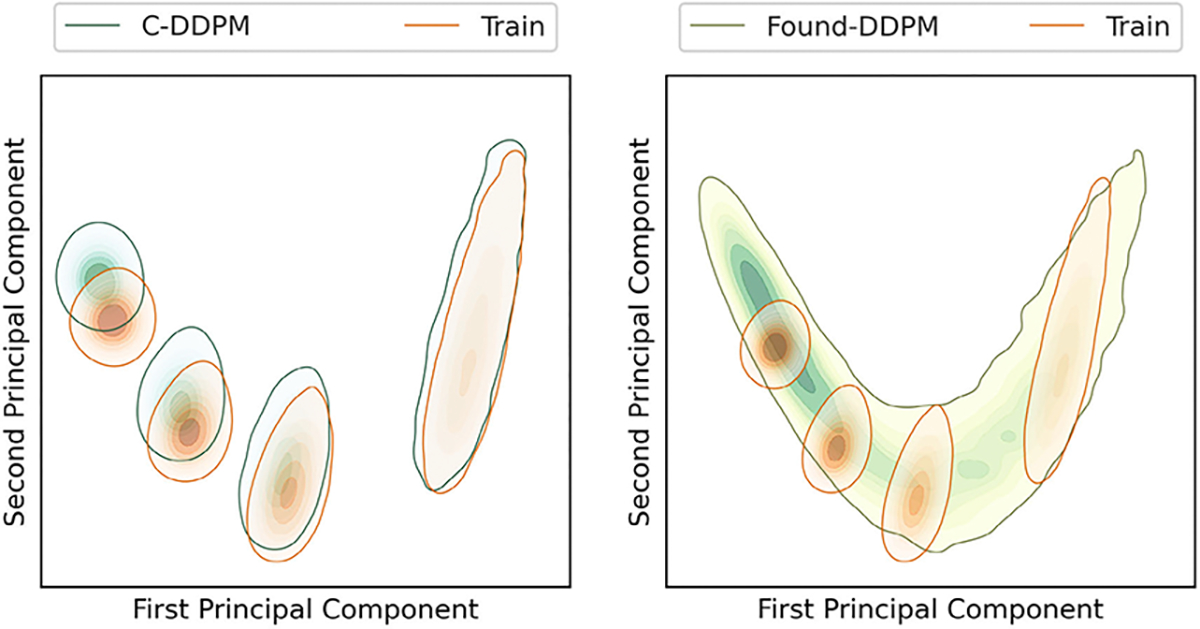
Results from the V-SCM for DDPM variants demonstrated via principal component analysis (PCA). Class-conditional DDPM (left) respects the four distinct modes in the data, but demonstrates unequal intra-class coverage and extrapolation. Foundational DDPM (right) performs very similar to the unconditional DDPM (Fig. 8) and provides slightly better coverage for some classes. Note that the PCA plots are represented via kernel density estimation of the data for display.

To assess if class-conditioning could alleviate class interpolation and extrapolation observed in DDPM, two class-conditioned DDPMs were trained on the V-SCM and the F-SCM. For both cases, the generated images were visually very similar to the unconditional DDPM images. In the first case (V-SCM), class-conditioning seemed to ensure that the distinct classes in the training data were retained well and interpolation between classes was absent. The class-specific implicit contextual features were also well replicated as observed in Fig. 14. However, similar to the unconditional DDPM (see Fig. 8), the exact distribution of the training data was not matched, albeit in a class-specific manner. Thus, class-conditioning may aid only in retaining distinct modes in the data as identified by labels, but not the exact class range.

In the second case (F-SCM), the foreground patterns that are indicative of class were assessed. As opposed to results from the unconditional DDPM, where forbidden foreground regions were not respected due to class interpolation in some cases, the conditional DDPM-generated ensemble never violated this rule. This supports the finding in case of the V-SCM that labeled classes are largely respected by a class-conditioned DDPM.

#### 2) Foundational DDPM:

Two foundational DDPMs (Found-DDPM) pre-trained on the ImageNet dataset were employed on the V-SCM and VT-SOM datasets to assess if they performed better than the vanilla DDPM. Recall that Found-DDPMs were afforded the same compute budget for fine-tuning as that afforded to the vanilla DDPM trained from scratch on our datasets. Visual quality of the V-SCM images generated from the Found-DDPM (not shown) was on par with those from the DDPM. For the Found-DDPM trained on V-SCM, it was observed that 9% of the realizations from the foundational DDPM demonstrated a Spearman rank-correlation *ρ* < 0.9, as compared to 4% from the unconditional DDPM ensemble. This indicates that about twice as many images in the Found-DDPM ensemble had *lower* quantitative fidelity than those in the vanilla DDPM ensemble. Furthermore, the reproducibility of implicit context in the DGM-generated images from the V-SCM was only slightly better for Found-DDPM as compared to vanilla DDPM in terms of class coverage as seen in Fig. 14 (right). Similar to the vanilla DDPM, the Found-DDPM demonstrated excellent mode coverage but did not respect the uniform class prevalence in the training data. Thus, in case of the V-SCM, both Found-DDPM and DDPM achieved similar results despite Found-DDPM having an advantage over DDPM in terms of pre-training and model capacity.

**Fig. 16.**
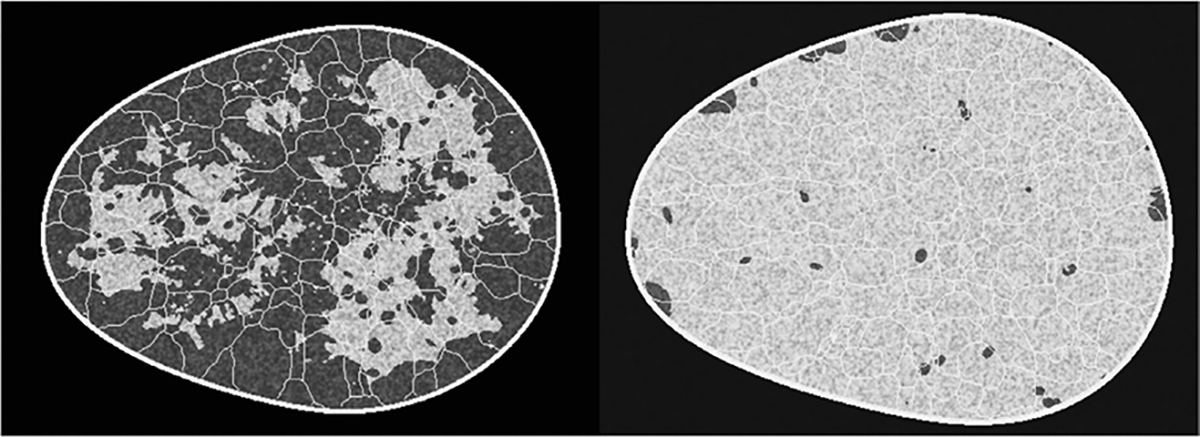
A visually good image (left), and an unrealistic image (right) in terms of ligament structure and extreme F/G ratio, generated from the Found-DDPM fine-tuned on the VT-SOM are shown. The visual image quality of these images is slightly lower than those generated from the vanilla DDPM.

For the VT-SOM, the visual quality of images generated from the Found-DDPM was slightly inferior to those generated from the DDPM. An example each of a visually high quality image, and an unrealistic image, from the Found-DDPM ensemble are shown in Fig. 16. This qualitative result was also reflected in the quantitative results described in Table V for all feature families and class-based analyses. However, we note that training Found-DDPM beyond the specified compute budget improved its performance to approximately match the vanilla DDPM (results not shown). Thus, the generalization capacity of the Found-DDPM might be constrained by the similarity between the datasets employed for pre-training and fine-tuning, as expected. Hence, in some cases, a vanilla DDPM might provide superior performance at lower computational cost. We discuss this result further in the Discussion section.

## V. Discussion

An evaluation of the DDPM via stochastic models of context provides insights into the generative capacity of the DDPM relevant to biomedical imaging, beyond conventional measures of image quality. Although contextual errors have been known to occur in the GAN family of DGMs [21], [37], [38], [39], to our knowledge, this is the first work to demonstrate and quantify various contextual errors in a diffusion-based generative model. Results from our studies demonstrate that impactful errors likely are present in every DGM-generated ensemble. The impact of those errors is task-dependent and therefore should be studied case-by-case. The relevance of the evaluation approach to medical imaging applications employed in this work lies not in anatomical realism, but in the logically analogous representation of contextual attributes relevant to biomedical imaging. For example, one work [39] reported contextual errors in GAN-generated images such as misplaced pacemakers in chest radiographs. Analogously, we observed misplaced tiles in DDPM-generated Flags-SCM images, thus exposing the capacity of DDPMs to misplace features in forbidden areas. Other examples of biomedical imaging scenarios that involve the studied contextual attributes include: (i) pathology images, wherein the cell-specific size, intensity distribution and per-image prevalence may be characteristic of different pathologies and (ii) the relative positions of organs, and the per-image prevalence of ribs in a chest radiograph. These examples are respectively analogous to: (i) the Voronoi SCM, which encodes context via shading and prevalence at multiple length scales, and (ii) the Alphabet SCM, which encodes context via per-image prevalence and relative positions of letters.

One key finding is that implicit context *for new classes* was very well reproduced in the DDPM-generated Voronoi SCM ensemble (see Fig. 8). This is particularly important because the implicit context that defines a genuine realization is known for Voronoi diagrams [56]. Thus, the “correctness” of a V-SCM realization actually can be measured. The corollary is that new experiments can be designed to determine the amount and type of training data required to generate *N* images of a desired quality, as is necessary to validate data augmentation schemes. This is in stark contrast to traditional measures of DGM-generated image quality which do not necessarily indicate at all the quality of image *content*. For example, there is no known set of statistics or formulas that defines what a human heart ought to look like; and the extent to which natural human perception impacts quality assessment of images is unknown, and may well be unquantifiable [41]. Thus, SCMs provide a domain-apt ground truth where none otherwise is known. The particular SCMs employed in the present work were chosen for ease of inspection and discussion. The key property that a designed SCM should have is post-hoc recoverability of context. This can be achieved in numerous ways but perhaps the simplest is to selectively constrain an existing stochastic model of interest. This is what was done to the VICTRE model. That model already was demonstrated to be anatomically adequate for some virtual imaging trials [43]. By imposing an exact distribution on intensity, a known correlation length on the arrangement of those intensities, and a slightly processed ligament structure, several recoverable contexts were created without losing the overall anatomical realism.

A second result, also pertaining to interpolation across classes, was observed via the F-SCM. Specifically, interpolated instances in the DDPM-generated ensemble were not merely a linear combination of foregrounds in the training data. Furthermore, some instances also violated the regions forbidden as foreground across all classes. However, the grid in the F-SCM design, or size of a single tile, was correctly learned by the DDPM, and it seems that this knowledge was employed in class interpolation. Thus, although the DDPM correctly identified the relevant local scale in the formation of patterns, it did not perfectly capture the image-level context in the F-SCM. On a more complex dataset: the VT-SOM, the DDPM largely failed to capture all contextual constraints at once. Despite the high visual quality of the DDPM images, errors in ligament formation were identified even by a non-domain-expert in about 30% of the ensemble. Furthermore, unrealistic extrapolation beyond all classes in the training data was observed in the V-SCM and F-SCM, potentially due to the likelihood-based approach of the DDPM that also contributes to excellent mode coverage [49]. Unlike the DDPM, the latent diffusion model employed on the V-SCM and VT-SOM, seemed to retain distinct classes. Especially, in the case of V-SCM (see Fig. 7), all four classes were distinctly formed but classes with low number of regions were preferentially generated. It is possible that the latent encoding of the LDM captures class information, constraining not just class interpolation but potentially also intra-class diversity as observed in the results from the VT-SOM. We also explored the possibility that a class-conditioned DDPM may alleviate the issue of class interpolation. The class-conditioned DDPM retained distinct classes but demonstrated the same effects (mismatched class-specific distribution) for *each* class that an unconditional DDPM did over the entire data distribution. This implies that class-conditioning may only avoid drastic interpolation in classes as determined by the labels, but not necessarily within a class or even in attributes unrelated to class labels.

**Fig. 17.**
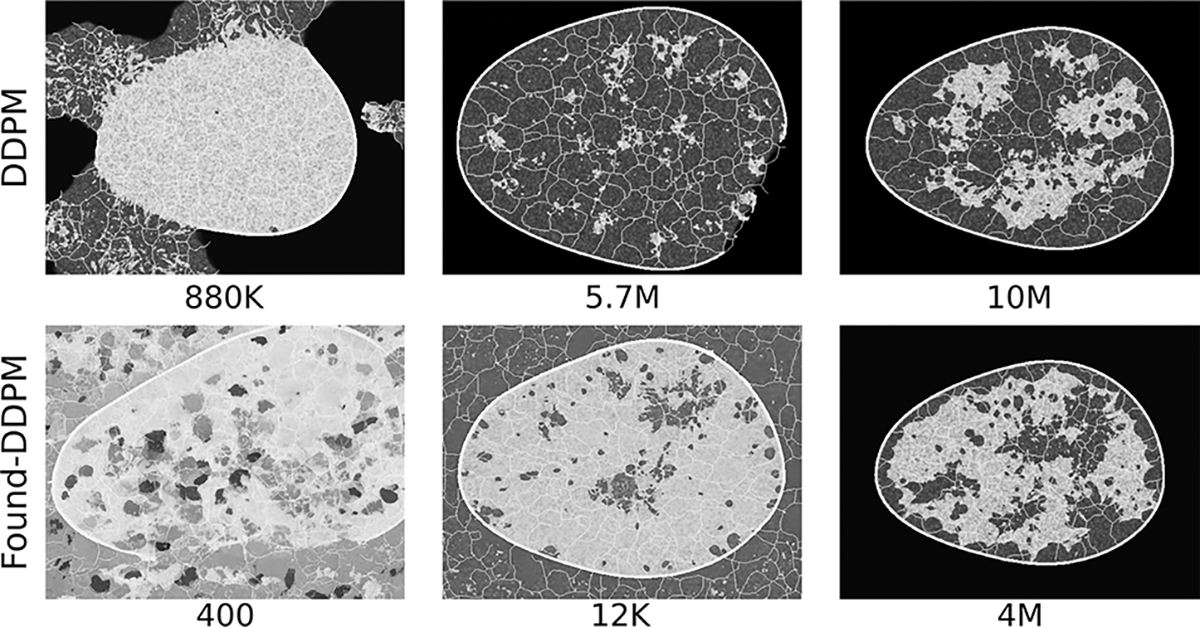
Visually interesting but random examples from the training trajectories of the DDPM and Found-DDPM models employed on VT-SOM. The training step corresponding to each image is indicated below the image, and represents the number of images seen in training. The DDPM seemed to shape clearly demarcated zero-valued background and foreground textures, while Found-DDPM seemed to unlearn placing textures in patches or all over the image before learning the expected features.

Another important factor highlighted through our results is the relation between a model’s intended use and the datasets employed for training, i.e., DGM generalizability. Our experiments demonstrated that although foundational DDPMs provide a powerful alternative to the vanilla DDPM, they may actually perform worse than the latter in some cases. In case of the VT-SOM, although Found-DDPM and DDPM both had the same compute budget, the Found-DDPM may have spent part of training unlearning ImageNet features before learning VT-SOM features. This is consistent with the training trajectories observed in Fig. 17. Possibly, Found-DDPM first had to learn that breast slices occupy only the central portion of an image and all textural features are contained within the egg-like shape of the slice, unlike the ImageNet where texture features may be translation invariant and the entire image is often non-zero. On the other hand, the vanilla DDPM seemed to learn to create a distinct zero-valued background early in training, followed by learning shape and texture at once. SG-XL did not perform well on the VT-SOM, potentially due to the projections into the ImageNet feature space involved in training. This lack of DGM generalizability was also observed in some of our experiments (not reported) with the F-SCM and with modern GANs such as BigGAN (unconditional version) [58], StyleGAN3 [59], SG-XL [48], all of which were especially difficult to train on this dataset. We hypothesize that the reasons may be: the dissimilarity of this SCM from the ImageNet dataset/ natural images (for SG-XL and BigGAN), features conditioned on pixel locations (for SG3), the prescribed random pixel placements, and the feature-specific intensity distributions in the F-SCM. We intend to explore these effects in our future work. Thus, novelty in DGM design aimed at improving performance on natural image synthesis may not provide the same potential benefits for medical imaging tasks, and hence, domain and task relevant evaluation remains critical before deploying state-of-the-art DGMs in medical imaging workflows.

**Fig. 18.**
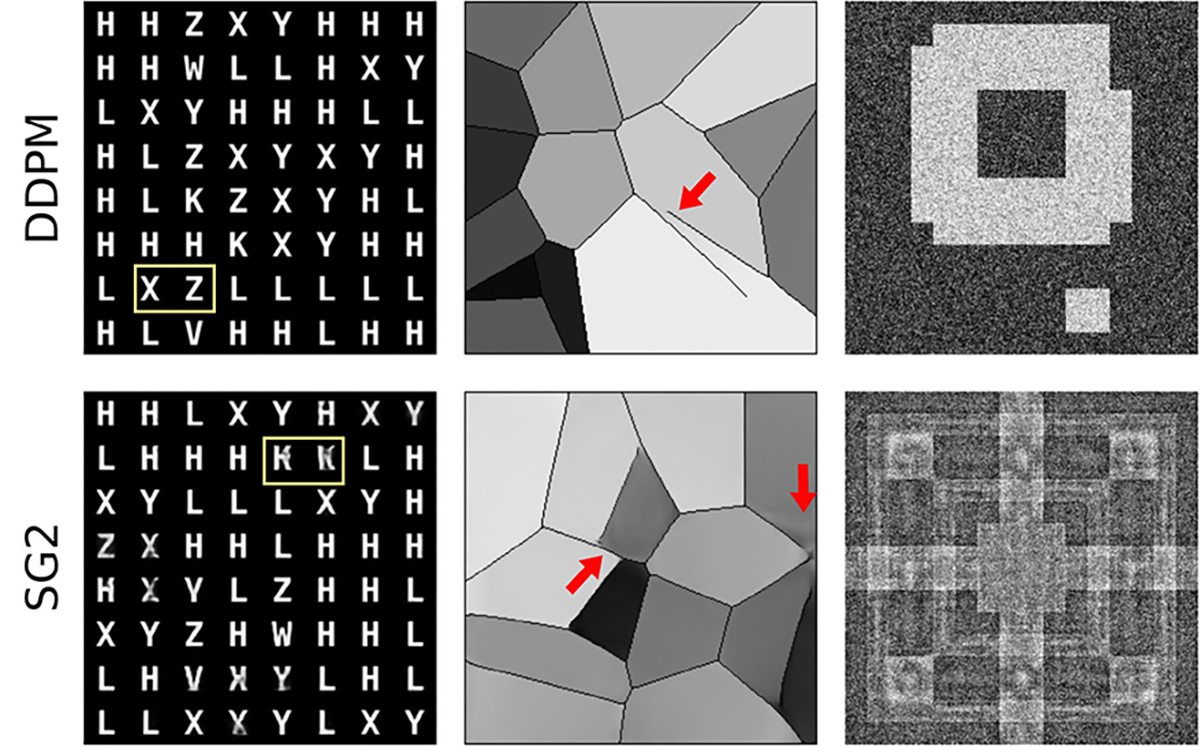
Examples of artifacts present in generated realizations from the DDPM and SG2 are shown for the three SCMs. Errors in DDPM-generated images demonstrate misplaced but distinct motifs from the training data, whereas errors in SG2-generated images demonstrate malformations or blending of distinct motifs. This effect is seen across all SCMs.

**Fig. 19.**
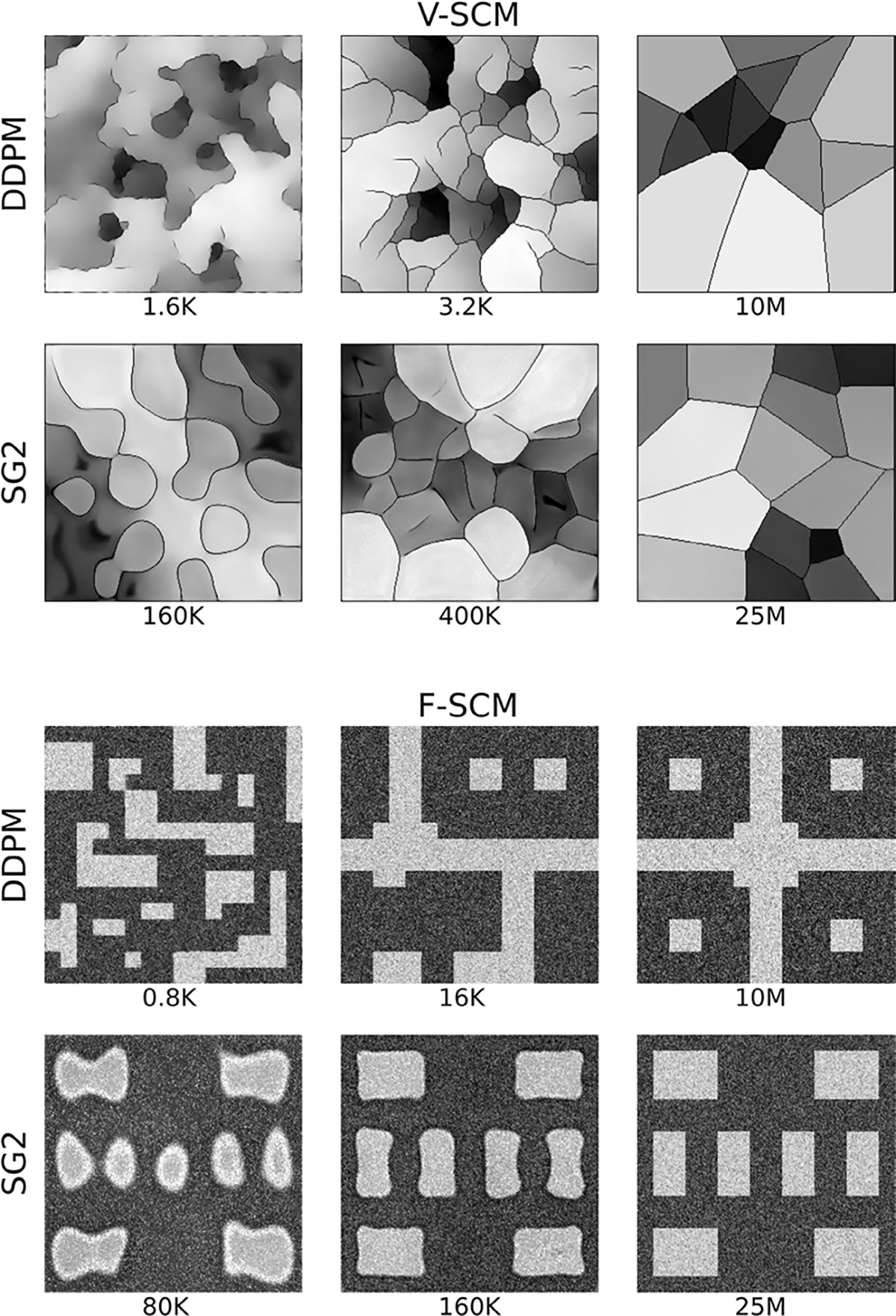
Visually interesting but random examples from the training trajectories of the DDPM and SG2 models employed on V-SCM and F-SCM datasets are shown. The training step corresponding to each image is indicated below the image, and represents the number of images seen in training. The DDPM seemed to first learn local elements that constitute the expected structure, while SG2 seemed to learn image structure by moulding blob-like elements.

Even the simple SCMs employed in this work reveal stark differences in image representation within DGM paradigms. For example, differences in the characteristic image representation of the DDPM and StyleGAN2 were observed, particularly, via the nature of the artifacts (see Fig. 18) and training trajectories Fig. 19. While artifacts in the DDPM generally seemed to involve misplaced but correct motifs, the artifacts in SG2 demonstrated a blending of various motifs within the same image. Similar differences were observed in the training trajectories (Fig. 19). The DDPM seemed to first learn local elements required to construct image-level structure followed by combinations of these elements, while SG2 seemed to learn image structure through blob-like elements.

A main motivation for our work is that, at this time in the field, evaluation on real data is necessary but challenging. This is chiefly because there is no ground truth to which to compare a newly generated image. We encoded contexts in our training data to serve as a kind of a ground truth and specifically to test whether it “survives” the generation process. The rationale is that if a given DGM cannot reproduce generic contexts which are well-known to be in many sorts of biomedical images, then that same DGM is unlikely to reproduce the even more sophisticated contexts found in real data. In this sense, although we cannot claim or guarantee general applicability, our results intuitively are applicable to a wider variety of DGMs than would they would be had we attempted to choose or model specific tissues or anatomy. Each SCM in this work represents a different context; together, the SCMs constitute a readily interpretable and intuitive method for the objective assessment of DGMs. The SCMs successively encode an increasing number of contextual constraints; this enables a step-wise evaluation of the capacity of a DGM to reproduce individual and joint contextual constraints (see Table I). For example, the DDPM almost perfectly replicates the letter prevalences in the A-SCM, and largely reproduces the contextual constraints of shading and prevalence in the V-SCM, but fails to reproduce the intensity distributions in the F-SCM. This suggests that the joint replication of multiple contextual constraints remains a challenge for DDPM.

## VI. Conclusion

In this work, an instance of the denoising diffusion probabilistic model (DDPM) was evaluated to gain insights into its capacity to reproduce contextual attributes analogous to anatomical constraints present in medical imaging scenarios. The DDPM-generated ensembles in this study demonstrated low contextual error-rates, but none of the ensembles reproduced the expected context perfectly. This evaluation goes beyond earlier evaluations of the DDPM that employed ensemble-based evaluation measures designed for natural images, or conventional measures of image quality. We anticipate that the employed evaluation framework might yield insights into emerging DGMs and have a broader impact on decision-making and DGM benchmarking.

## Figures and Tables

**Fig. 1.**
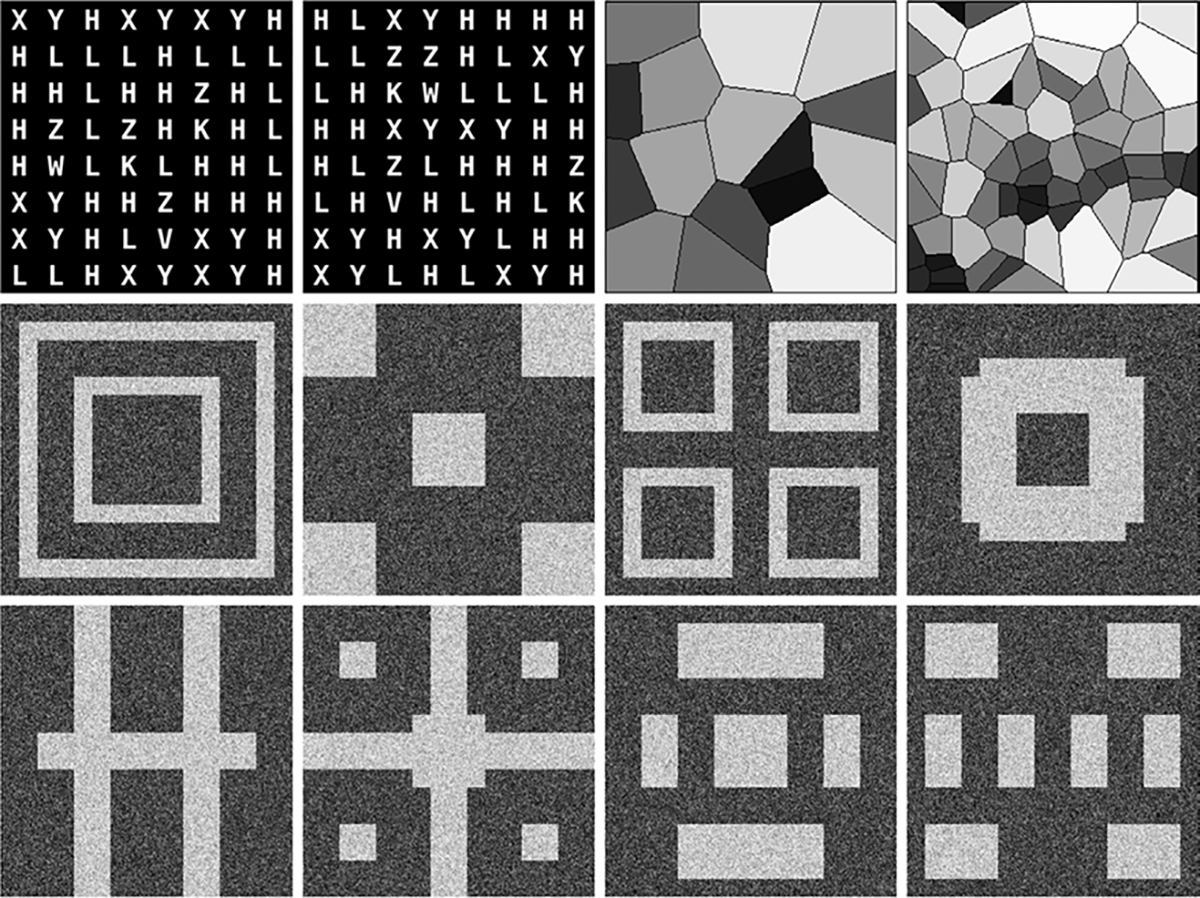
Sample realizations from all three SCMs are shown. Top row: Two realizations each from the single-class A-SCM (left) and the four-class V-SCM (right). Realizations from the V-SCM represent classes 16 and 64 respectively. Rows 2 and 3: A realization from each of the eight classes in the F-SCM.

**Fig. 2.**
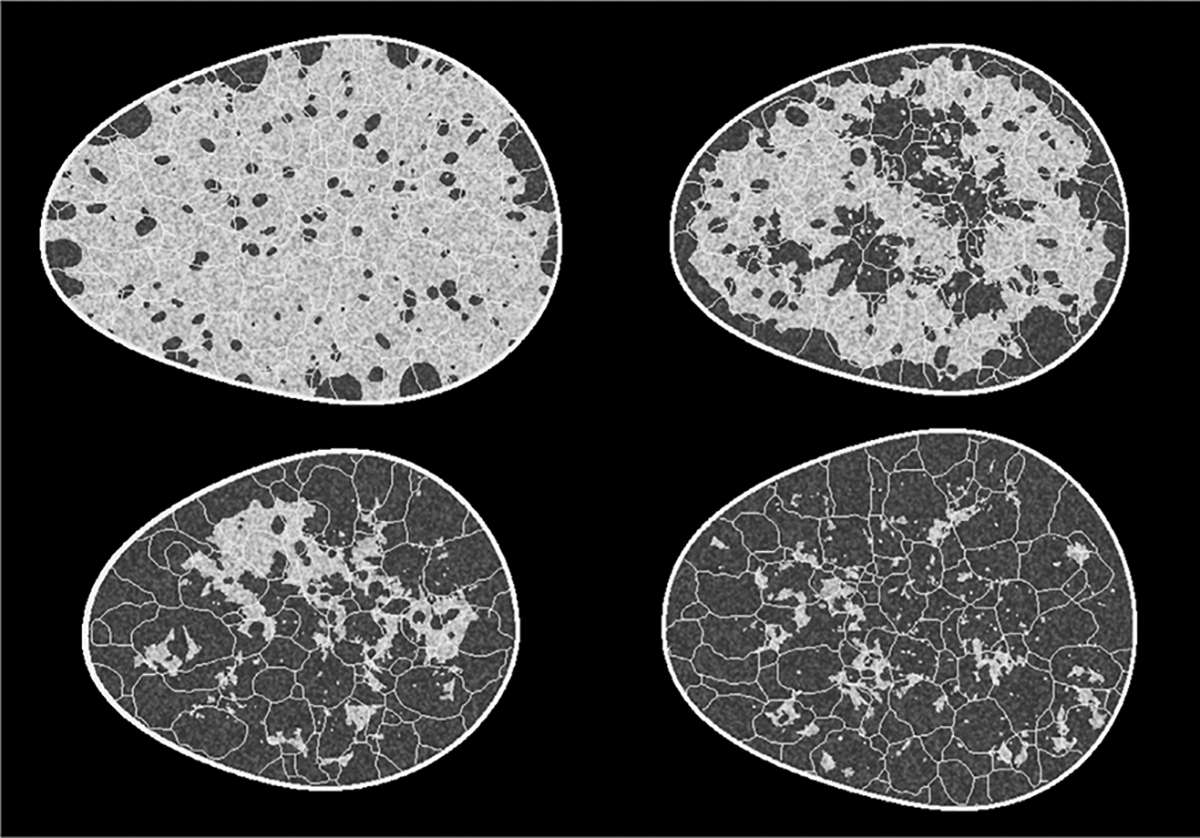
Sample images from each of the four classes in the VT-SOM. Sample realizations from (top row: L to R) dense, heterogeneous, (bottom row: L to R) scattered, and fatty breast types are shown.

**Fig. 15.**
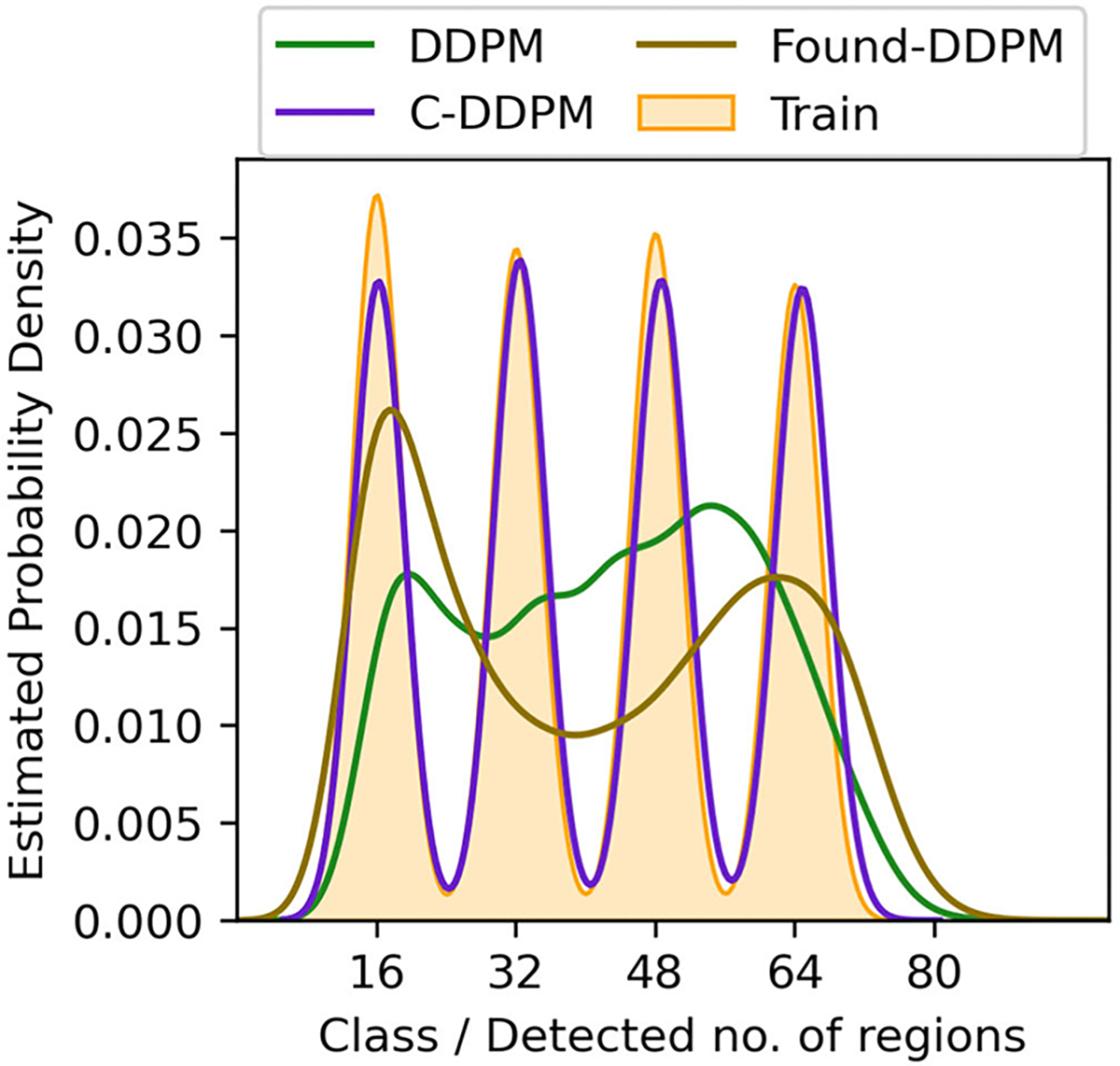
Class-prevalence results from the V-SCM for DDPM variants demonstrated via kernel density estimates. Class-conditional DDPM (C-DDPM) demonstrates an excellent match with the training data in terms of class modes and prevalence. However, foundational DDPM (Found-DDPM) demonstrated similar effects: mode coverage, class interpolation and extrapolation, as compared to the unconditional vanilla DDPM.

**TABLE I:** Overview of All Stochastic Context and Object Models in Terms of the per-Image Contextual Constraints Explicitly Prescribed in the Model

Constraints	A-SCM	V-SCM	F-SCM	VT-SOM

Prevalence	✓	✓	✓	✓
Intensity	X	✓	✓	✓
Texture	X	✓	✓	✓
Position	✓	X	✓	✓
Anatomy	X	X	X	✓
Multi-class	X	✓	✓	✓

**TABLE II:** Results From the F-SCM. Percentage of Acceptable Realizations in an Ensemble Is Reported for All DGMs and for Four Contextual Constraints. The DDPM Slightly Outperforms SG2 in Most Cases. Results for Prevalence and Position Are Reported Together Because These Constraints Jointly Define the Foreground Structure Representative of a Class

Constraints	Measure of error	DDPM		SG2	
		FG	BG	FG	BG

Prevalence + Position	RMAE	99	99	98	98
Intensity	*χ* ^2^	0	0	0	9
Texture	Moran’s I	100	99	96	95

**TABLE III:** Results From the VT-SOM for Various Feature Families. Most Feature Families Were Better Reproduced in the DDPM-Generated Ensemble as Compared to the Other DGM-Generated Ensembles, as Indicated by the Lower KS Statistic for the Former. (Skeleton Statistics Were Not Computable for SG-XL Because of Badly Formed Skeletons.

Feature set / DGM	DDPM	SG2	LDM	SG-XL

	KS statistic			
Texture features	**0.028**	0.062	0.085	0.123
Morphology features	**0.049**	0.280	0.129	0.255
Skeleton statistics	**0.006**	0.190	0.102	NA
F/G ratio	0.230	**0.110**	0.227	0.159
Overall	**0.028**	0.270	0.140	0.312*

* Indicates That Skeleton Statistics Were Excluded in This Computation

**TABLE IV:** Analysis of Class Prevalence, Coverage and Density Demonstrate the Superior Performance of the DDPM in Representing All Classes Present in the Training Data. The Class-Wise Prevalence in the Training Data Was: 10%, 40%, 40%, 10%.

DGM	Class prevalence (%)	Class coverage [55]	Class density [55]

DDPM	21,44,29,6	0.97, 0.96, 0.91, 0.91	0.99, 1.01, 0.98, 1.02
LDM	11,26,34,29	0.82, 0.59, 0.60, 0.49	0.98, 1.00, 0.92, 0.55
SG2	6,43,45,6	0.35, 0.53, 0.74, 0.60	1.02, 0.82, 0.99, 1.02
SG-XL	12,34,23,31	0.01, 0.07, 0.07, 0.14*	0.00, 0.05, 0.11, 0.13*

* Indicates That Skeleton Statistics Were Excluded in This Computation

**TABLE V:** Results From the VT-SOM for Foundational DDPM. In All Cases, the Vanilla DDPM Outperformed Found-DDPM, Given a Fixed Compute Budget. However, We Observed That Additional Training of the Found-DDPM Brought Its Performance at Par With the Vanilla DDPM (Results Not shown)

Feature set / DGM	DDPM	Found-DDPM

	KS statistic	
Texture features	**0.028**	0.134
Morphology features	**0.049**	0.085
Skeleton statistics	**0.006**	0.049
F/G ratio	**0.230**	0.460
Overall	**0.028**	0.069

Class prevalence(%)	21,44,29,6	41,37,20,2
Class coverage [55]	0.97, 0.96, 0.91, 0.91	0.81, 0.89, 0.64, 0.57
Class density [55]	0.99, 1.01, 0.98, 1.02	0.59, 0.98, 0.76, 1.01

## References

[1] Kazeminia S , “GANs for medical image analysis,” Artif. Intell. Med, vol. 109, Sep. 2020, Art. no. 101938. 34756215 10.1016/j.artmed.2020.101938

[2] Shamsolmoali P , “Image synthesis with adversarial networks: A comprehensive survey and case studies,” Inf. Fusion, vol. 72, pp. 126–146, Aug. 2021.

[3] Kazerouni A , “Diffusion models in medical imaging: A comprehensive survey,” Med. Image Anal, vol. 88, Aug. 2023, Art. no. 102846. 37295311 10.1016/j.media.2023.102846

[4] Khader F , “Denoising diffusion probabilistic models for 3D medical image generation,” Sci. Rep, vol. 13, no. 1, p. 7303, May 2023. 37147413 10.1038/s41598-023-34341-2 PMC10163245

[5] Dorjsembe Z, Odonchimed S, and Xiao F, “Three-dimensional medical image synthesis with denoising diffusion probabilistic models,” in Proc. Med. Imag. Deep Learn, 2022, pp. 1–3.

[6] You C , “Structurally-sensitive multi-scale deep neural network for low-dose CT denoising,” IEEE Access, vol. 6, pp. 41839–41855, 2018. 30906683 10.1109/ACCESS.2018.2858196 PMC6426337

[7] Lyu Q, You C, Shan H, Zhang Y, and Wang G, “Super-resolution MRI and CT through GAN-CIRCLE,” Proc. SPIE, vol. 11113, pp. 202–208, Sep. 2019.

[8] You C , “CT super-resolution GAN constrained by the identical, residual, and cycle learning ensemble (GAN-CIRCLE),” IEEE Trans. Med. Imag, vol. 39, no. 1, pp. 188–203, Jan. 2020. 10.1109/TMI.2019.2922960 PMC11662229 31217097

[9] You C, Yang J, Chapiro J, and Duncan JS, “Unsupervised Wasserstein distance guided domain adaptation for 3D multi-domain liver segmentation,” in Interpretable and Annotation-Efficient Learning for Medical Image Computing, Lima, Peru. Cham, Switzerland: Springer, 2020, pp. 155–163.

[10] You C , “Class-aware adversarial transformers for medical image segmentation,” in Proc. Adv. Neural Inf. Process. Syst, vol. 35, 2022, pp. 29582–29596. PMC10395073 37533756

[11] Bond-Taylor S, Leach A, Long Y, and Willcocks CG, “Deep generative modelling: A comparative review of VAEs, GANs, normalizing flows, energy-based and autoregressive models,” IEEE Trans. Pattern Anal. Mach. Intell, vol. 44, no. 11, pp. 7327–7347, Nov. 2022. 34591756 10.1109/TPAMI.2021.3116668

[12] Song Y and Ermon S, “Generative modeling by estimating gradients of the data distribution,” in Proc. Adv. Neural Inf. Process. Syst, vol. 32, 2019, pp. 1–13.

[13] Song Y, Sohl-Dickstein J, Kingma DP, Kumar A, Ermon S, and Poole B, “Score-based generative modeling through stochastic differential equations,” 2020, arXiv:2011.13456.

[14] Song Y, Shen L, Xing L, and Ermon S, “Solving inverse problems in medical imaging with score-based generative models,” 2022, arXiv:2111.08005.

[15] Karras T, Laine S, Aittala M, Hellsten J, Lehtinen J, and Aila T, “Analyzing and improving the image quality of StyleGAN,” in Proc. IEEE/CVF Conf. Comput. Vis. Pattern Recognit. (CVPR), Jun. 2020, pp. 8110–8119.

[16] Sohl-Dickstein J, Weiss E, Maheswaranathan N, and Ganguli S, “Deep unsupervised learning using nonequilibrium thermodynamics,” in Proc. Int. Conf. Mach. Learn., 2015, pp. 2256–2265.

[17] Pinaya WHL , “Brain imaging generation with latent diffusion models,” 2022, arXiv:2209.07162.

[18] Ho J, Jain A, and Abbeel P, “Denoising diffusion probabilistic models,” in Proc. NIPS, vol. 33, 2020, pp. 6840–6851.

[19] Nichol AQ and Dhariwal P, “Improved denoising diffusion probabilistic models,” in Proc. 38th Int. Conf. Mach. Learn., vol. 139, Jul. 2021, pp. 8162–8171.

[20] Dhariwal P and Nichol A, “Diffusion models beat GANs on image synthesis,” in Proc. NIPS, vol. 34, 2021, pp. 8780–8794.

[21] Müller-Franzes G , “Diffusion probabilistic models beat GANs on medical images,” 2022, arXiv:2212.07501.

[22] Jalal A, Arvinte M, Daras G, Price E, Dimakis AG, and Tamir J, “Robust compressed sensing MRI with deep generative priors,” in Proc. Adv. Neural Inf. Process. Syst, vol. 34, 2021, pp. 14938–14954.

[23] Chung H and Ye JC, “Score-based diffusion models for accelerated MRI,” Med. Image Anal, vol. 80, Aug. 2022, Art. no. 102479. 35696876 10.1016/j.media.2022.102479

[24] Liu J , “DOLCE: A model-based probabilistic diffusion framework for limited-angle CT reconstruction,” 2022, arXiv:2211.12340.

[25] Huy PN and Quan TM, “Denoising diffusion medical models,” 2023, arXiv:2304.09383.

[26] Iskandar M , “Towards realistic ultrasound fetal brain imaging synthesis,” 2023, arXiv:2304.03941.

[27] Heusel M, Ramsauer H, Unterthiner T, Nessler B, and Hochreiter S, “GANs trained by a two time-scale update rule converge to a local nash equilibrium,” in Proc. Adv. Neural Inf. Process. Syst, vol. 30, 2017, pp. 1–12.

[28] Salimans T, Goodfellow I, Zaremba W, Cheung V, Radford A, and Chen X, “Improved techniques for training GANs,” in Proc. Adv. Neural Inf. Process. Syst, vol. 29, 2016, pp. 1–9.

[29] Zhou W, Bovik AC, Sheikh HR, and Simoncelli EP, “Image quality assessment: From error visibility to structural similarity,” IEEE Trans. Image Process, vol. 13, pp. 600–612, 2004. 15376593 10.1109/tip.2003.819861

[30] Woodland M , “Feature extraction for generative medical imaging evaluation: New evidence against an evolving trend,” 2023, arXiv:2311.13717. 10.1007/978-3-031-72390-2_9 PMC12117514 40444256

[31] Borji A, “Pros and cons of GAN evaluation measures: New developments,” Comput. Vis. Image Understand, vol. 215, Jan. 2022, Art. no. 103329.

[32] Borji A, “Pros and cons of GAN evaluation measures,” Comput. Vis. Image Understand, vol. 179, pp. 41–65, Feb. 2019.

[33] Usman Akbar M, Wang W, and Eklund A, “Beware of diffusion models for synthesizing medical images - a comparison with GANs in terms of memorizing brain MRI and chest X-ray images,” 2023, arXiv:2305.07644.

[34] Usman Akbar M, Larsson M, and Eklund A, “Brain tumor segmentation using synthetic MR images - a comparison of GANs and diffusion models,” 2023, arXiv:2306.02986. 10.1038/s41597-024-03073-x PMC10904731 38424097

[35] Chuquicusma MJM, Hussein S, Burt J, and Bagci U, “How to fool radiologists with generative adversarial networks? A visual Turing test for lung cancer diagnosis,” in Proc. IEEE 15th Int. Symp. Biomed. Imag. (ISBI), Apr. 2018, pp. 240–244.

[36] Park HY , “Realistic high-resolution body computed tomography image synthesis by using progressive growing generative adversarial network: Visual Turing test,” JMIR Med. Informat, vol. 9, no. 3, Mar. 2021, Art. no. e23328. 10.2196/23328 PMC8077702 33609339

[37] Deshpande R, Anastasio MA, and Brooks FJ, “A method for evaluating deep generative models of images via assessing the reproduction of high-order spatial context,” 2021, arXiv:2111.12577.

[38] Kelkar VA , “Assessing the ability of generative adversarial networks to learn canonical medical image statistics,” IEEE Trans. Med. Imag, vol. 42, no. 6, pp. 1799–1808, Jun. 2023. 10.1109/TMI.2023.3241454 PMC10314718 37022374

[39] Schütte AD , “Overcoming barriers to data sharing with medical image generation: A comprehensive evaluation,” Npj Digit. Med, vol. 4, no. 1, pp. 1–14, Sep. 2021. 34561528 10.1038/s41746-021-00507-3 PMC8463544

[40] Oliva A and Torralba A, “The role of context in object recognition,” Trends Cognit. Sci, vol. 11, no. 12, pp. 520–527, Dec. 2007. 18024143 10.1016/j.tics.2007.09.009

[41] Emrith K, Chantler MJ, Green PR, Maloney LT, and Clarke ADF, “Measuring perceived differences in surface texture due to changes in higher order statistics,” J. Opt. Soc. Amer. A, Opt. Image Sci., vol. 27, no. 5, pp. 1232–1244, 2010. 10.1364/JOSAA.27.001232 20448792

[42] Doersch C, Gupta A, and Efros AA, “Context as supervisory signal: Discovering objects with predictable context,” in Computer Vision—ECCV, Zurich, Switzerland. Cham, Switzerland: Springer, 2014, pp. 362–377.

[43] Badano A , “Evaluation of digital breast tomosynthesis as replacement of full-field digital mammography using an in silico imaging trial,” JAMA Netw. Open, vol. 1, no. 7, Nov. 2018, Art. no. e185474. 30646401 10.1001/jamanetworkopen.2018.5474 PMC6324392

[44] Deshpande R, Anastasio MA, and Brooks FJ, “Stochastic context models designed for the evaluation of deep generative models of images,” Harvard Dataverse, Tech. Rep., 2021, doi: 10.7910/DVN/HHF4AF.

[45] Sauvola J and Pietikäinen M, “Adaptive document image binarization,” Pattern Recognit, vol. 33, no. 2, pp. 225–236, Feb. 2000.

[46] Deep Generative Modeling for Learning Medical Image Statistics (Dgm-Image Challenge). Accessed: Apr. 15, 2023. [Online]. Available: https://www.aapm.org/GrandChallenge/DGM-Image/

[47] Liberman L and Menell JH, “Breast imaging reporting and data system (BI-RADS),” Radiologic Clinics North Amer, vol. 40, no. 3, pp. 409–430, May 2002. 10.1016/s0033-8389(01)00017-3 12117184

[48] Sauer A, Schwarz K, and Geiger A, “StyleGAN-XL: Scaling StyleGAN to large diverse datasets,” in Proc. Special Interest Group Comput. Graph. Interact. Techn. Conf, Aug. 2022, pp. 1–10.

[49] Xiao Z, Kreis K, and Vahdat A, “Tackling the generative learning trilemma with denoising diffusion GANs,” in Proc. Int. Conf. Learn. Represent., 2022, pp. 1–28.

[50] Simonyan K and Zisserman A, “Very deep convolutional networks for large-scale image recognition,” 2014, arXiv:1409.1556.

[51] Nunez-Iglesias J, Blanch AJ, Looker O, Dixon MW, and Tilley L, “A new Python library to analyse skeleton images confirms malaria parasite remodelling of the red blood cell membrane skeleton,” PeerJ, vol. 6, p. e4312, Feb. 2018. 29472997 10.7717/peerj.4312 PMC5816961

[52] van der Walt S , “Scikit-image: Image processing in Python,” PeerJ, vol. 2, p. e453, Jun. 2014. 25024921 10.7717/peerj.453 PMC4081273

[53] Haralick RM, Shanmugam K, and Dinstein I, “Textural features for image classification,” IEEE Trans. Syst., Man, Cybern, vol. SMC-3, no. 6, pp. 610–621, Nov. 1973.

[54] Chakravarti IM, Laha RG, and Roy J, Handbook of Methods of Applied Statistics. New York, NY, USA: Wiley, 1967.

[55] Naeem MF, Oh SJ, Uh Y, Choi Y, and Yoo J, “Reliable fidelity and diversity metrics for generative models,” in Proc. Int. Conf. Mach. Learn., 2020, pp. 7176–7185.

[56] Boots B, Sugihara K, Chiu SN, and Okabe A, Spatial Tessellations: Concepts and Applications of Voronoi Diagrams. Hoboken, NJ, USA: Wiley, 2009.

[57] Moran PAP, “Notes on continuous stochastic phenomena,” Biometrika, vol. 37, no. 1, p. 17, Jun. 1950. 15420245

[58] Brock A, Donahue J, and Simonyan K, “Large scale GAN training for high fidelity natural image synthesis,” in Proc. 7th Int. Conf. Learn. Represent., 2019, pp. 1–35.

[59] Karras T , “Alias-free generative adversarial networks,” in Proc. Adv. Neural Inf. Process. Syst, vol. 34, 2021, pp. 852–863.

